# Circulating nuclear DNA structural features, origins, and complete size profile revealed by fragmentomics

**DOI:** 10.1172/jci.insight.144561

**Published:** 2021-04-08

**Authors:** Cynthia Sanchez, Benoit Roch, Thibault Mazard, Philippe Blache, Zahra Al Amir Dache, Brice Pastor, Ekaterina Pisareva, Rita Tanos, Alain R. Thierry

**Affiliations:** 1 IRCM, Institut de Recherche en Cancérologie de Montpellier, INSERM U1194, Université de Montpellier, Institut régional du Cancer de Montpellier, Montpellier, France.; 2 Thoracic Oncology Unit, Arnaud de Villeneuve Hospital, University Hospital of Montpellier, Montpellier, France.

**Keywords:** Genetics, Oncology, Cancer, Diagnostics, Pharmacogenetics

## Abstract

To unequivocally address their unresolved intimate structures in blood, we scrutinized the size distribution of circulating cell-free DNA (cfDNA) using whole-genome sequencing (WGS) from both double- and single-strand DNA library preparations (DSP and SSP, *n* = 7) and using quantitative PCR (Q-PCR, *n* = 116). The size profile in healthy individuals was remarkably homogenous when using DSP sequencing or SSP sequencing. CfDNA size profile had a characteristic nucleosome fragmentation pattern. Overall, our data indicate that the proportion of cfDNA inserted in mono-nucleosomes, di-nucleosomes, and chromatin of higher molecular size (>1000 bp) can be estimated as 67.5% to 80%, 9.4% to 11.5%, and 8.5% to 21.0%, respectively. Although DNA on single chromatosomes or mono-nucleosomes is detectable, our data revealed that cfDNA is highly nicked (97%–98%) on those structures, which appear to be subjected to continuous nuclease activity in the bloodstream. Fragments analysis allows the distinction of cfDNA of different origins: first, cfDNA size profile analysis may be useful in cfDNA extract quality control; second, subtle but reliable differences between metastatic colorectal cancer patients and healthy individuals vary with the proportion of malignant cell-derived cfDNA in plasma extracts, pointing to a higher degree of cfDNA fragmentation and nuclease activity in samples with high malignant cell cfDNA content.

## Introduction

The analysis of circulating cell-free DNA (cfDNA) undoubtedly represents a breakthrough in the diagnostic field (1–4). The potential of this newly identified source of biological information has attracted the attention of researchers and clinicians in numerous fields (3–5). CfDNA sizing has emerged as a new strategy in the optimization of cfDNA analysis.

Because of the high nuclease sensitivity of the naked DNA molecule, the size of the extracted cfDNA is intimately associated with the biological particle structure transporting and stabilizing it. Consequently, in recent years these 2 features have been highly scrutinized, to improve knowledge of cfDNA release, to improve cfDNA detection, and to evaluate cfDNA potential to discriminate cfDNA tissue/cells of origin, with the aim of increasing cfDNA diagnostic power (6–10). CfDNA can exist as protein-associated DNA fragments, or can lie in extracellular vesicles, within the physiological circulating fluids of both healthy and diseased individuals (2, 3). CfDNA is derived not only from genomic DNA but also from extrachromosomal mitochondrial DNA (11). Even though cfDNA has presently an increasing number of clinical applications (1, 12), its structural characteristics have yet to be fully elucidated.

CfDNA was initially thought to be up to 40 kb in size, but principally 180 bp (or multiples of), corresponding to the size of the DNA packed in a mono-nucleosome (13, 14). Observations of mono- and oligo-nucleosomes led to the view that the major mechanism of cfDNA release is apoptosis (2, 14, 15). Using Q-PCR to examine fragment size, we initially demonstrated that (a) cfDNA is highly fragmented (16, 17); (b) cfDNA quantification is more efficient at lower amplicon sizes; and (c) cfDNA fragments can be as small as 45 bp (9, 18). Furthermore, we established that the lower the size of the detected cfDNA amplicon (down to 60–70 bp), the higher the quantified amount (16). Since that observation, all Q-PCR primer systems specifically designed for detecting cfDNA have now been designed to detect amplicons smaller than 100 bp, or, optimally, smaller than 80 bp (5, 6, 19–21).

However, cfDNA fragment size distribution obtained by Q-PCR was significantly different than that obtained by next-generation sequencing, showing a major population peaking at 166 to 167 bp. Q-PCR revealed high levels of fragmentation, with most of the fragments found below 145 bp in the plasma from both healthy and cancer patients (6, 8, 16, 18, 22, 23). Although no single current method for analyzing cfDNA size profile is optimal, previous reports have only used 1 method at a time. This has made it difficult to obtain a precise and unequivocal overall cfDNA size profile. In a blinded study, we previously observed that cfDNA from cancer patients has a similar size distribution, whether using Q-PCR or nonconventional whole-genome deep sequencing (WGS) from a single-strand DNA library preparation (SSP, ref. 6). In contrast to the standard WGS from double-strand DNA library preparation (DSP), SSP sequencing (SSP-S) revealed a significant proportion of short cfDNA fragments (below 80 bp); this was something not readily detectable by DSP sequencing (DSP-S), as previously shown by Underhill et al. (10). This provided new insights into cfDNA size profiles and harmonized sequencing and Q-PCR findings (16).

Previous deep sequencing examination of cfDNA fragmentation patterns revealed that they are specific signatures of tissue origins, that short cfDNA fragments harbor footprints of nucleosomes as well as transcription factors, and that cfDNA from healthy individuals derives from hematopoietic cells (8). Higher fragmentation has been found in the cfDNA of cancer patients (16), in tumor cells (9), and in the fetal fraction (24, 25). Efforts are ongoing to increase analytical sensitivity in this area, by focusing on a specific cfDNA fragment size range. CfDNA fragmentation analysis is also being pursued as a possible means of stratifying individuals (9, 26–30).

In our study, we used the synergistic analytical information provided by Q-PCR and by WGS of both double- and single-stranded DNA libraries in order to unequivocally observe cfDNA size distribution in healthy subjects. This enabled us to measure cfDNA size precisely over a wide range of lengths and thus obtain information about DNA strand degradation and detectable cfDNA structures. We also performed the following 2 comparisons, in a blinded fashion: first, using WGS (DSP-S and SSP-S), we precisely compared the size profile up to approximately 1000 bp of cfDNA obtained from 7 healthy individuals and 7 metastatic colorectal cancer patients; second, using Q-PCR, we compared the size fraction distribution of cfDNA in the wide range length in plasma obtained from 109 healthy individuals and 104 colorectal cancer (CRC) patients.

## Results

### Circulating plasma DNA size profiling by whole-genome sequencing.

**Figure 1:**
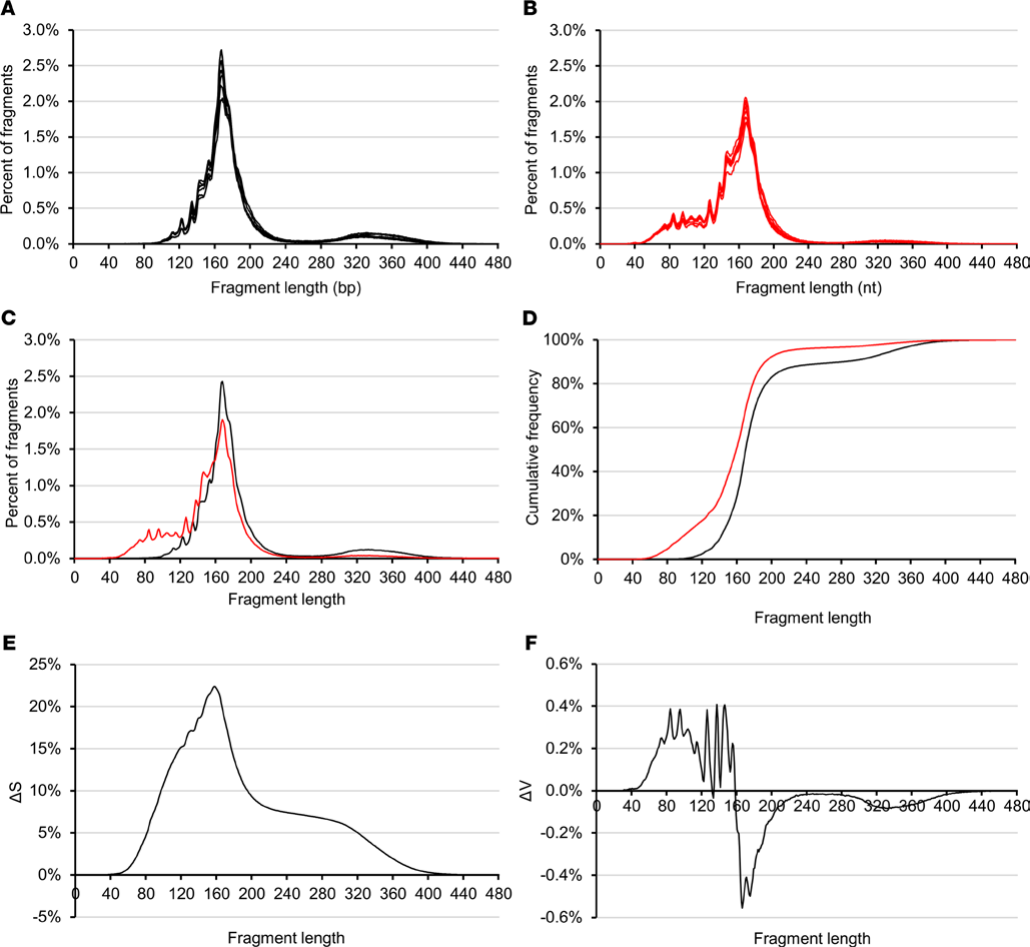
CfDNA size profile as determined from DSP-S and SSP-S. CfDNA size profiles of 7 healthy individuals, obtained by sequencing either from double- or single-strand DNA library preparations (**A** and **B**, respectively). Mean size profiles of the 7 individuals, as determined by DSP-S (black lines) and SSP-S (red lines) (**C**); curves of the cumulative frequencies between SSP-S and DSP-S (**D**); the difference in cumulative frequencies, denoted as ΔS, between SSP-S minus DSP-S (**E**); and the curve of the difference of % values, denoted as ΔV, between SSP-S minus DSP-S (**F**). The increasing part of the ΔS curve indicates the fragment size range, in which SSP-S detected fragment number is proportionally higher than DSP-S detected fragments; while the decreasing part of the ΔS curve indicates the fragment size range in which SSP-S–detected fragment number is proportionally lower than for DSP-S–detected fragments (**E**). Positive ΔV values for cfDNA size indicate where more fragments were detected by SSP-S than by DSP-S (**F**). Negative ΔV values for cfDNA size indicate where less fragments were detected by SSP-S than by DSP-S. More fragments are detected by SSP-S up to 158 bp (nt) as compared with DSP-S, and more fragments are detected by DSP-S over 158 bp (nt) (**F**).

Sequencing libraries are prepared from either DSP or SSP. Both methods provide profiles from which variations can be detected and compared, with cfDNA sizes ranging from approximately 30 to approximately 1000 bp/nt (6, 31). Figure 1 shows size profiles of cfDNA from 7 healthy human individuals obtained by both DSP-S and SSP-S. For all the samples, we obtained a mean of 1,434,487 reads (1,079,717–1,611,205 reads) for DSP and 1,007,070 reads (963,701–1,299,291 reads) for SSP (Supplemental Figure 1; supplemental material available online with this article; https://doi.org/10.1172/jci.insight.144561DS1). Size profiling of cfDNA from the 7 plasma samples revealed very low variation, as all 7 curves superimposed, irrespective of the DSP-S or SSP-S libraries (Figure 1, A and B).

The DSP-S cfDNA profile of healthy subjects had a major monomodal population between 80 and 260 bp, peaking at 166 bp with approximately 2.5% of total fragments (Figure 1A). A smaller population was also detectable between 260 and 420 bp, ranging from 8.0% to 12.9% of the total fragments (Figure 1, A and B, and Supplemental Table 1A). Subpeaks of the 7 samples colocalized (Table 1 and Supplemental Table 1B). Reliable reads were detectable down to 40 bp in most of the samples.

The SSP-S cfDNA size profile of healthy subjects had a population between 45 and 260 bp, which peaked at 166 bp, corresponding to approximately 2.0% of the total fragments. Fragments plateaued between 70 and 120 bp at approximately 0.4% (Figure 1B). A very small population was observed between 250 and 400 nt that ranged from 2.7% to 4.5 % of the total fragment number (Supplemental Table 1). All subpeaks of the 7 samples colocalized (Table 1, Table 2, and Supplemental Table 1B). Reliable reads were detectable down to the limit of the sequencing detection, approximately 25 nt.

As determined by DSP-S and SSP-S, the cfDNA size profile showed clear discrepancies. Regarding the fraction ranging up to 90 bp, or from 90 to 240 bp, or from 240 to approximately 440 bp, the proportions of cfDNA averaged 0.1%, 87.2%, and 12.7%, respectively, as determined by DSP-S; and 8.0%, 87.2%, and 4.8%, respectively, as determined by SSP-S (Figure 1). Fragments shorter than 80 bp (nt) were only detectable by SSP-S (Figure 1C and Supplemental Table 2). Between 80 and 166 bp (nt), the proportion of cfDNA fragments determined by single-strand DNA sequencing was slightly higher than for double-strand DNA sequencing: 56.9% and 41.5%, respectively (Supplemental Table 2A). Conversely, SSP-S values were slightly lower in the 166 to 240 bp (nt) range, constituting 33.1% of the total number of fragments, as compared with the DPS-S values, which constituted 45.5%. It is not possible to compare the number of reads in SSP-S and DSP-S size profiles, but the respective proportions within any size range is informative (Supplemental Figure 1).

We directly compared the performance of the 2 sequencing techniques by scrutinizing data obtained from difference in cumulative frequencies, denoted as ΔS, or the difference of fragment frequency, denoted ΔV (Supplemental Methods, Appendix 1). Altogether, data showed that SSP-S enabled the detection of a higher number of cfDNA fragments as compared with DSP-S and revealed a higher number of fragments in the 45 to 158 bp (nt) range and a lower number of fragments in ranges from 158 to 250 bp (nt) and, to a lesser extent, 280 to 440 bp (nt).

While a major cfDNA peak and a very minor peak were observable at approximately 166 bp and approximately 320 bp (Figure 1, A and B), there were also subpeaks every approximately 10 bp, due to the intimate structure of cfDNA and its association with histone octamers. Tables 1 and 2 summarize the detection of these subpeaks in healthy individual cfDNA, from either SSP or DSP. The different sequencing techniques produced differences in subpeaks at specific cfDNA sizes. The differences between the library preparations included subpeaks at 53, 63, 73, 83, and 94 bp that were only observed with SSP-S, and not with DSP-S; whereas a subpeak at 152 bp was only seen with DSP-S. No periodicity was detected between 145 and 167 bp when using SSP-S (Tables 1 and 2). Note also that the SSP-S–derived subpeaks are approximately 3 bp higher than those of DSP-S.

### Size distribution analysis by Q-PCR.

**Figure 2:**
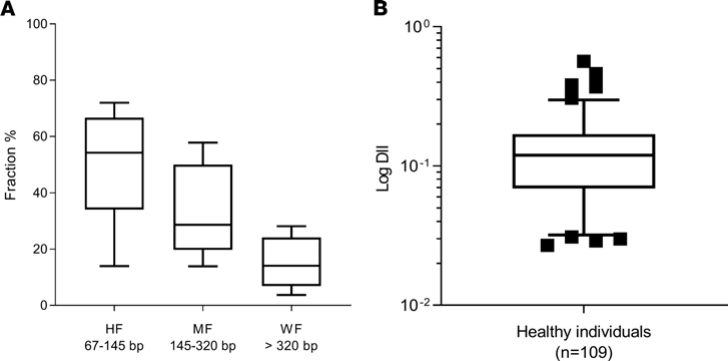
CfDNA size distribution as determined by Q-PCR. Fractional size distribution was performed using nested Q-PCR primer systems to detect amplicons of 67, 145, and 320 bp in the 7 healthy individuals (Supplemental Methods, Appendix 2). Note, fractional size distribution as presented here was obtained from cfDNA concentrations quantified by targeting the *KRAS* DNA region and is only indicative, as described in the Methods section. The cfDNA size distribution was summarized by presenting the levels (data represent mean ± SEM) in the highly fragmented cfDNA fraction (HF, 67–145 bp), the levels in the mono-nucleosome–derived fragmented cfDNA (MF, 145–320 bp), and a lower proportion (3%–20%) in the weakly fragmented cfDNA (WF, >320 bp). (**A**). The DNA Integrity Index (DII) was calculated based on the Q-PCR–based determination of the ratio of the number of fragments over 320 bp to those over 67 bp within a *KRAS* intron 3/exon 2 region in a panel of 109 healthy individuals (**B**). The sample median DII was 0.119. Bar, median; box, 25% to 75%; brackets, 5% to 95%; see *Statistics* section in Methods.

Next, we used nested Q-PCR primer systems to detect amplicons of 67, 145, and 320 bp, to estimate the proportion of the different cfDNA size fractions in the 7 samples (Figure 2A). (Note: cfDNA concentrations as reported here concern a *KRAS* DNA region, and are only indicative of the total cfDNA concentration, as indicated in the Methods section.) This technique could detect cfDNA down to 67 bp. The highly fragmented cfDNA fraction (HF, 67–145 bp) and the mono-nucleosome–derived cfDNA fragment fraction (MF, 145–320 bp) were detected in similar proportions (38% and 39%, mean, respectively), whereas the weakly fragmented fraction (WF, >320 bp) was found in a lower proportion (23%; Figure 2A). To corroborate our findings, we tested a panel of 109 healthy individuals (Figure 2B). The sample mean DNA Integrity Index (DII) was 0.134 ± 0.091 SD (Supplemental Figure 2), indicating that, for fragments over 67 bp, approximately 13.4% are over 320 bp; this confirms the WF fraction (19%) derived from the 7 samples mentioned above.

### Comparison of the size profile of plasma cfDNA from healthy subjects and subjects with metastatic CRC.

There was very little variation in the size profiles of cfDNA from the healthy individuals determined by each of the 2 sequencing methods (Figure 1A). Because of this, we used the mean size profile for healthy subjects as our reference in the remainder of this study, when comparing the fragmentation of cfDNA from cancer and healthy plasma.

**Figure 3:**
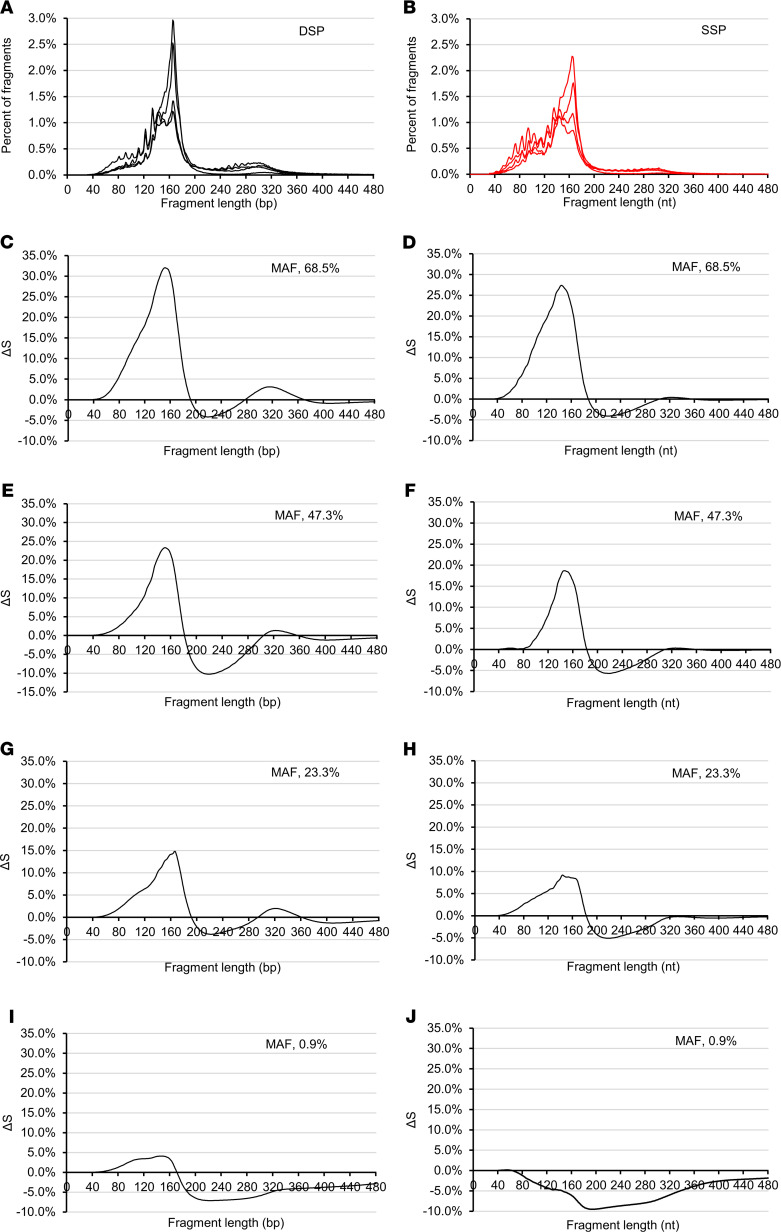
CfDNA size profile from 4 illustrative patients with metastatic CRC. Sequencing from double- or single-stranded DNA library preparations (**A** and **B**, respectively). The difference in cumulative size frequencies, denoted as ΔS, between individual cancer samples and healthy DNA mean as determined by DSP-S (**C**, **E**, **G**, and **I**) or SSP-S (**D**, **F**, **H**, and **J**). MAF of metastatic CRC (mCRC) patients: 68.5% (**C** and **D**), 47.3% (**E** and **F**), 23.3% (**G** and **H**), and 0.9% (**I** and **J**). The individual size profiles and cumulative size frequency curves from each mCRC patient are presented in Supplemental Figures 3, 4, 6, and 7.

There were subtle but reliable differences between the DSP-S size profiles of each of the 7 cancer patients and the mean of the 7 healthy individuals (Supplemental Figure 3). Although the cfDNA fragment populations of both cancer and healthy subjects peaked at 166 bp, cancer patient plasma had more cfDNA fragments between 40 and 150 bp and less between 150 and 260 bp. Also, the profile curve showed a shoulder between 145 and 166 bp in cancer patient plasma (Figure 3, A, C, E, G, and I; and Supplemental Figure 3).

The similar subtle but reliable differences were observed by SSP-S (Figure 3, B, D, F, H, and J; and Supplemental Figure 4); cancer patient plasma had more fragments between 30 and 145 nt and less between 145 and 260 nt, while both populations peaked at 166 nt (Figure 3). Note, the shoulder observed in cancer patients (145–160 nt) was slightly more pronounced with SSP-S, as can be seen by comparing the size profile of the cfDNA with the highest mutant allele frequency (MAF) (Supplemental Figure 4).

**Figure 4:**
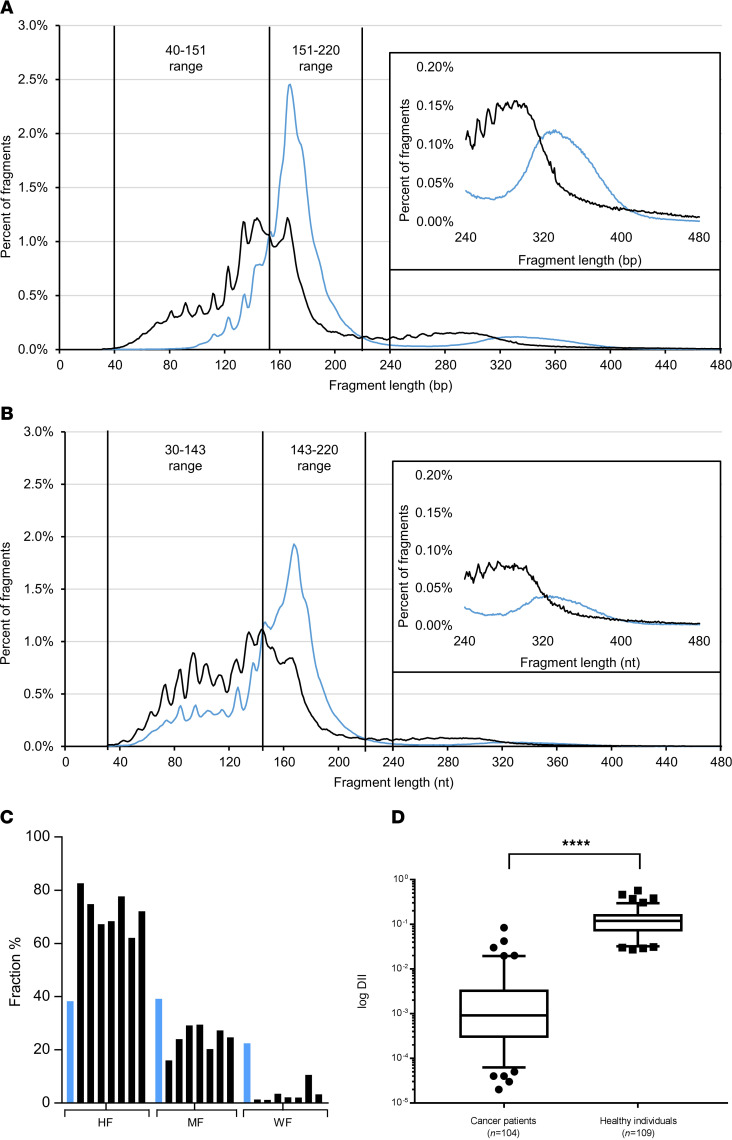
Comparison of the cfDNA size distribution of healthy individuals and mCRC patients. Comparison of the cfDNA size profile of the healthy individual mean (blue line) and a cancer patient with a MAF of 68.5% (black line), as determined by DSP-S (**A**) and SSP-S (**B**). Vertical lines indicate the fragment lengths, where the size profile curve of healthy mean cfDNA crosses that of cancer patient cfDNA. Insert, zoom on the 240–480 bp (nt) size range. Size distribution, as determined by Q-PCR analysis from mean of the 7 healthy individuals (blue) and 7 cancer (black), of the HF (67–145 bp), MF (145–320 bp), and WF (>320 bp) fractions (**C**). Note, fractional size distribution as presented here was obtained from cfDNA concentrations quantified by targeting the *KRAS* intron 3 region and is only indicative, as described in the Methods section. DII as determined by calculating the ratio of the WF fraction over total cfDNA concentration (>67 bp) within a *KRAS* intron 3 DNA region (**D**). Bar, median; box, 25% to 75%; brackets, 5% to 95%. The level of significance was assessed by Student’s *t* test.

CRC patient number 8 had the highest MAF (69%). Juxtaposing their DSP-S or SSP-S cfDNA size profile with the mean healthy line illustrated the overall differences in cfDNA fragmentation between healthy subjects and mCRC patients (Figure 4, A and B). Using DSP-S to make the same comparison, the respective size profile curves differed greatly. The curves of the DSP-S and SSP-S size profiles from cancer plasma appeared to be shifted to lower size, while peaking at the same size (166 bp) when compared with the curves from healthy individuals (Figure 4, A and B). As observed in Figure 4, A and B, as well as in ΔV curves (Figure 1F and Supplemental Figure 5), the difference in frequency between cancer and healthy subjects by DSP-S was positive in the 40 to 150 and 220 to 320 bp (nt) ranges and negative in the 150 to 220 bp range (Supplemental Figure 5); for SSP-S, it was positive in the 30 to 140 and 220 to 320 bp (nt) ranges and negative in the 140 to 220 bp (nt) range (Supplemental Figure 5).

Whether detected by DSP-S or SSP-S, the differences between healthy and cancer subjects increased with the MAF in all mCRC samples (Figure 3 and Supplemental Figures 2, 3, and 5). Note, the higher the MAF, the greater the number of shorter fragments and the smaller the peak at 166 bp. The mean fraction of cfDNA fragments whose size corresponded to that of cfDNA fragments packed in the di-nucleosome structure, was 8% to 12.9 % and 2.5% to 4.5% in healthy plasma, and 3.2% to 17.9% and 1.5% to 9.5% in cancer patients (Supplemental Table 1), with DSP-S and SSP-S, respectively. In contrast, the di-nucleosome–associated peak was significantly different in healthy and cancer patient plasma (~332 vs. approximately 300 bp, and approximately 327 vs. approximately 303 nt, as detected by DSP-S and SSP-S, respectively). As derived from DSP-S analysis, the 166 bp/145 bp fragment size frequency ratio showed discriminative power between the healthy samples (3.1 ± 0.33 SD) and the 7 mCRC samples (1.0 to 3.29; Supplemental Table 2A). Using SSP-S analysis, the 166 bp/145 bp fragment size frequency ratio was 1.58 ± 0.10, and ranged from 0.77 to 2.05 in the mean healthy samples and the 7 CRC samples, respectively (Supplemental Table 2A). Moreover, using DSP-S analysis, the fragment size frequency of the 30 to 145 bp range, as compared with the total fragment size in the 30 to 440 bp range (corresponding to DNA in mono- and di-nucleosomes), showed discriminative power between the healthy samples (13.40 ± 0.02 SD) and the 7 CRC samples (17.35 to 44.05). Using SSP-S analysis, the fragment size frequency of the 30 to 145 bp range was 33.08 ± 0.02 and ranged from 28.05 to 60.38 in the mean healthy samples and the 7 CRC samples, respectively (Supplemental Table 2A).

**Figure 5:**
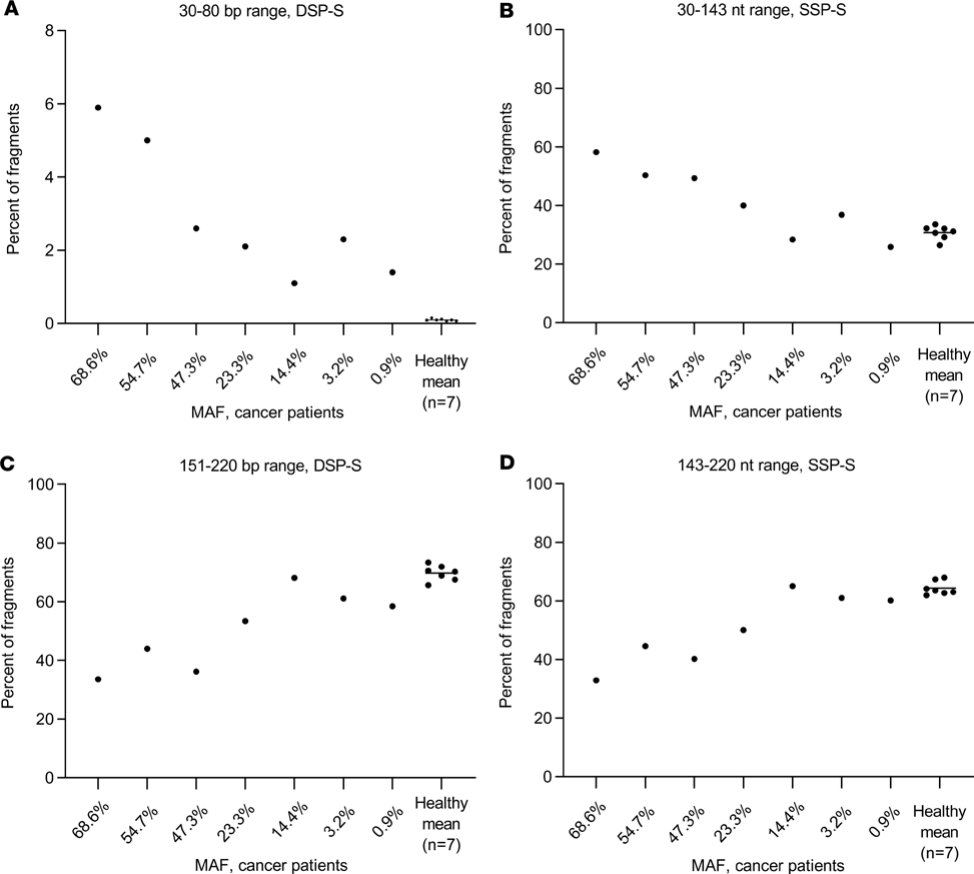
Illustration of the capacity of fragmentomics in distinguishing cfDNA released from healthy and malignant cells. Fragment percentage as determined by DSP-S (**A** and **C**) and SSP-S (**B** and **D**) in the 30–80 bp (**A**), 30–143 nt (**B**), 151–220 bp (**C**), and 143–220 nt (**D**) size ranges in the total cfDNA fragment population from healthy individual mean (*n* = 7) and from single cancer patients of various MAFs. The figure only presents the size range in which the cfDNA fragment proportion showed a the highest variation between the healthy mean and the patient with the highest MAF (68.6%).

The observation of the size distribution plot of cumulative size frequencies, and the ΔS, or ΔV, between individual cancer samples and the healthy cfDNA mean, enabled us to refine the difference between mean healthy and each cancer patient (Figure 3, Supplemental Table 2, and Supplemental Figures 5–7). Note, this difference at the peak increased as MAF increased (0.9%, 3.2%, 14.3%, 23.3%, 47.3%, 54.6%, and 68.5%). Thus, ΔS peak value difference was smallest for the mCRC cases with the lowest MAF, using either DSP-S (5%, 15%, 24%, and 34%) or SSP-S (0, 8, 18 and 26%; Figure 3 and Supplemental Figures 6 and 7). Nevertheless, the area under the ΔS curve appeared higher, when examining the size profile, in SSP-S than in DSP-S analysis (Figure 3 and Supplemental Figure 8). In addition, the ΔS at 155 bp (nt) varied from 3.9% to 31.80%, and from –5.70% to 24.9% (when using DSP-S and SSP-S, respectively), and appeared as a discriminatory factor when comparing cancer and healthy individuals (Supplemental Table 2B). Figure 5 illustrates that cfDNA fragment frequency at specific size ranges correlates with MAF. Fragment percentage of the 30–80 bp or 30–143 nt size range increased with elevated MAF as determined by DSP-S and SSP-S, respectively; fragment percentage of the 151–220 bp or 143–220 nt size range decreased with elevated MAF, as determined by DSP-S and SSP-S, respectively (Figure 5).

Similar observations can be made in relation to the calculation of ΔV. For both DSP-S and SSP-S analysis, the positive and negative ΔV curve peaks decreased with decreasing MAF, down to nearly no difference whatsoever (±0.2% at MAF = 0.9%, ΔV) (Supplemental Figure 5). Overall, data showed that the more MAF increased, the more observable differences there were in size profile and ΔS and ΔV curves (Supplemental Figure 5). When comparing cancer and healthy individual plasma, significant differences were observed in ΔS and ΔV data when DSP-S–derived values were subtracted from SSP-S–derived values (Supplemental Figures 5 and 8). In addition, the ΔV of the 40 to 160 bp (nt) size range varied from 3.32% to 29.96%, and from –6.13% to 22.05 %, when using DSP-S and SSP-S, respectively; ΔV also appeared as a discriminatory factor when comparing cancer and healthy individuals (Supplemental Figure 5 and Supplemental Table 2). Furthermore, ΔV calculated within the 40 to 160 bp (nt) range from the mean of 7 healthy plasma was 22.04 ± 0.68 SD %; and ΔV of the plasma from the 7 CRC patients varied from 12.27% to 16.68% (Supplemental Figure 5 and Supplemental Table 2B).

At specific cfDNA sizes, there were a number of differences in the presence of subpeaks between cancer and healthy individuals, depending on whether DSP-S or SSP-S analysis was used. These may be summed up as follows: DSP-S showed subpeaks at 71, 81, and 91 bp in cancer subjects (Supplemental Table 3) in contrast to healthy subjects (Table 1); SSP-S showed no subpeaks at approximately 150 nt in healthy subjects (Table 1), in contrast to cancer subjects (Supplemental Table 3).

The fractional size distribution determined by Q-PCR revealed that, in contrast to the plasma of healthy subjects, mCRC patient plasma samples showed a higher number of fragments in the HF than in the MF fraction and a very low level (~1%) in the WF fraction (Figure 4C). To corroborate our findings related to the DII, calculated for the 7 mCRC patients, we used a panel of 104 mCRC patients (Figure 4D and Supplemental Figure 2). In the CRC patients, the mean DII was 0.004. This means that 0.4% were higher than 320 bp and, since no fragments over that size are detectable up to ~1000 bp by WGS, that 0.4% were over approximately 1000 bp. Thus, the DII from the healthy cohort (mean DII, 0.13) was significantly higher than the DII from CRC patients of all stages (*P* < 0.0001; Figure 2 and Supplemental Figure 2).

## Discussion

Sizing by WGS allows the precise measurement of cfDNA fragments below approximately 1000 bp. Conventional DSP-S–derived size distribution relies on double-strand breaks in the DNA molecule, whereas size profiling by SSP-S can also reveal the level of nicks on both strands and can artificially measure single-stranded cfDNA fragments. CfDNA size distribution obtained from the conventional whole-genome sequencing of a double-stranded DNA library should be distinguished from that obtained from a single-stranded DNA library, or from Q-PCR; both use single-strand DNA as a first template (6). Consequently, collecting the information from DSP-S and SSP-S sizing provides clues about the cfDNA molecule positioning on the biological constituents (complexes) that stabilize them in the blood circulation. Size profiling using Q-PCR, on the other hand, shows the fractional size distribution (16, 18, 23, 32) and relies on “denatured” cfDNA fragments just as SSP-S relies on single-strand fragments (6); also, in contrast to WGS, Q-PCR allows analysis to be extended to lengths over approximately 1000 bp.

Given all of the elements detailed above, it will be obvious that the originality and the significance of our work, both in purely scientific terms and in its potential for clinical application, are that it combines Q-PCR, DSP-S, and SSP-S, and in doing so obtains an assessment of cfDNA size profile, fragmentation level, and associated structures that is simultaneously more complete and more precise.

### Size distribution of healthy donor cfDNA

Our first, surprising observation was that the size profile curves of the 7 healthy subjects were equivalent with each other, as were the 7 curves superimposed with either DSP-S or SSP-S. Consequently, we postulate that (a) the dynamics of DNA degradation following cell release is the same in all healthy subjects or (b) the resulting stabilized cfDNAs all have the same structure. Detailed analysis of cfDNA sizing revealed an approximately 10 bp (nt) periodicity footprint, which is detected down to 101 bp and 53 nt within the 41 to 166 bp (nt) range, using DSP-S and SSP-S, respectively. This suggests that nucleosome-derived degradation occurs once nuclear DNA/chromatin is released in blood. Thus, this pattern was attributed to cleavage in nucleotides, which are accessible because they lie further from the surface of the histone core at each helical turn where DNA wraps around the core (33). Consequently, our data confirm that most of the detectable cfDNA in blood has a nucleosome footprint (6–8, 10, 30); this indicates that the stability of circulating DNA derives mostly from the nucleosome structure. Although the number of cfDNA fragments associated with di-nucleosomes is relatively low, the 10 bp periodicity footprint is detectable within the 280–400 bp (nt) size range (with both DSP-S and SSP-S); that range corresponds to the length of DNA wrapped around a di-nucleosome. Indeed, recent reports suggest that the 2 key DNA/protein complexes that protect DNA from blood nucleases are probably DNA-wrapped around a histone octamer, or DNA-bound to transcription factors (6, 8, 34, 35). By generating maps of genome-wide in vivo nucleosome occupancy, Snyder et al. (8) revealed the presence of shorter (35–80 bp) fragments associated with cleavage adjacent to transcription factor–binding sites, harboring footprints of transcription factors (8, 36). It is likely that such transcription factor–associated cfDNA exists and that it may be present in a hidden manner, without being characterized in the size profile within the population of short cfDNA fragments. Our current study was not designed to individualize transcription factor–associated structures. SSP-S clearly revealed a population of short fragments and a more pronounced shoulder at approximately 145 bp, further revealing nicks in both strands of the DNA packed in the mono-nucleosome– or transcription factor–associated cfDNA. CfDNA associated with di-nucleosomes would therefore represent a very small proportion of the total cfDNA of healthy individuals.

**Figure 6:**
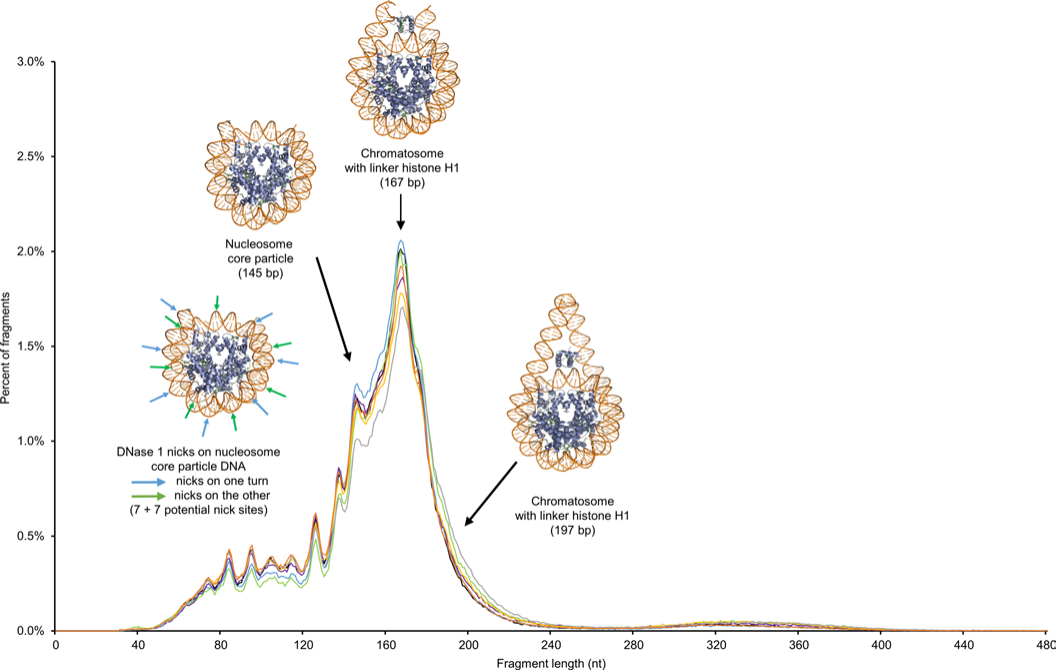
Representation of the crystal structure of the nucleosome core particle, chromatosome, and chromatosome with a flexible DNA chain, on the cfDNA fragment size profile of the 7 healthy subjects, as determined by SSP-S. The chromatosome with 167 bp DNA fragment is the most present cfDNA-associated structure, while being of low frequency (~2%). The nucleosome core particle devoid of H1 containing 147–160 bp is the second most present structure (1.1%–1.2%). Arrows on a nucleosome structure indicate the minor groove DNA sites subject to DNase attacks, explaining the ~10 bp periodic subpeaks in size profile revealing nicks on the nucleosome-associated DNA, and fragmentation down to 40 nt single-stranded DNA, when using SSP-S. Images of the crystal structure of chromatosome and nucleosome at 3.5 angstrom resolution, from the NIPDB data bank (4QLC and 5ONW, respectively). NIPDB, Nucleic Acid–Protein Interaction Database, https://npidb.belozersky.msu.ru

When using conventional Illumina Y adapters, we also assume that in the presence of double-stranded molecules with nick(s), only the strand without a nick will be recovered following DSP-S. In addition, DSP-S will reveal cfDNA fragments if there is 1 nick in both strands in the same vicinity. Furthermore, if there were 1 or 1+ nicks on each strand, and the double-stranded molecule still hung together, neither strand would be recovered by DSP-S; SSP-S, however, would detect as fragments *n*+1 number of single-stranded DNA pieces released from *n* nicks. Our data confirm that trimmed mono-nucleosome cfDNA-associated structures (theoretically condensing 165-bp length DNA) are predominant in the cfDNA size profile. Although WGS can only reveal the size profile from 30 to approximately 1000 bp, our data nevertheless distinctly demonstrate that the number and mass of cfDNAs within mono-nucleosomes is at least approximately 9 and approximately 4.5 times higher than the number and mass of cfDNAs associated with di-nucleosomes, respectively. Since both SSP-S and DSP-S gave the same peak at approximately 166 bp, we can hypothesize that a significant but low fraction (2–3%) of cfDNA fragments of this size are nick free, at least in 1 strand. This structure corresponded to the chromatosome, which consists of a histone octamer ([H2A–H2B]_2_ [H3-H4]_2_) plus the histone monomer linker H1 tightly associating 166 bp DNA (ref. 37 and Figure 6). Our data showed that the cfDNA molecule is highly nicked (97–98%) and that nuclease activity occurred in a continuous way on the nucleosomal structure. Thus, the nucleosome structure corresponding to the chromatosome devoid of the histone monomer linker H1 and then compacting only 147 bp DNA (the mono-nucleosome) was also highly represented among the cfDNA structural forms (Figure 6). DSP-S revealed nucleosomal footprints of cfDNA fragments smaller than 166 bp but did not reveal fragments smaller than 90 bp; this was in contrast to SSP-S analysis, which showed fragments as small as 45 nt. If fragments in the 45 to 90 nt range go undetected, this suggests that they are either degraded or no longer wrapped within the histone complex. Taken together, these observations imply that, in order to be protected, the cfDNA molecule needs to be surrounded by histones and as a result of this protection is detectable in blood samples. The 2 strands exposed to the surface of the nucleosome are shifted by 3 bp with a 3′ stagger. Indeed, our data showed a shift of 3 bp between the size of the molecules detected by DSP-S and SSP-S; in addition to the observation of the approximately 10 bp periodicity, this confirms that cfDNA are wrapped around the nucleosome (8).

Since the approximately 10 bp subpeaks were clearly observable within the 90 to 166 bp range, our data also suggest that most cfDNA molecules within that range derive from double-strand breaks occurring at the nucleosome extremity, as well as at 1 of the 14 positions on the DNA minor groove, where DNA is exposed at the nucleosome surface. It might be possible that rare double-strand breaks occur at 2 distinct positions at the nucleosome surface. Logically, double-strand breaks may occur at any one of the 14 positions (Figure 6). We should therefore have observed the same number of cfDNA fragments of each of the ~10, 20, 30, 40, 50, 60, 70, 80, 90, 100, 110, 120, 130, or 140 bp sizes. However, this was not the case; for instance, cfDNA fragments from 10 to 90 bp were not detected. The lower the DNA molecule size, the less tightly they are maintained on the nucleosome, as there are fewer binding forces. The disappearance of these fragments might result from their peeling off from the nucleosome and consequent rapid degradation. Reports have demonstrated that DNA may peel off from the edge of the nucleosome (37). This observation can therefore be taken as a convincing demonstration that the nucleosome is an essential element of cfDNA stability.

SSP-S revealed shorter cfDNA fragments, down to 45 nt, because the DNA molecule with only 1 nick on one of the 2 DNA molecule strands was still maintained and wrapped around the nucleosome and therefore did not peel off. The critical size of 70 bp corresponds to a full turn around the nucleosome. This suggests that if there is a DNA fragment associated with a less than full turn around the nucleosome, the probability of peeling off is high, as a consequence of its degradation. Figure 6 shows the position of the minor groove where nicks may occur and offers a schematic view of the nucleosome/chromatin structures associated with cfDNA, depending on cfDNA fragment length (Figure 6).

Theoretically, any fragment sizes detectable by DSP-S should also be detectable by SSP-S. In contrast to DSP-S analysis, no 152 bp subpeak was observable with SSP-S; however, this does not mean that this double-strand fragment was not present in the extract, rather that it was invisible because of its lower frequency, compared with that of the neighboring subpeaks, especially at 145 bp. Nevertheless, this highlights the fact that the 145 to 166 bp DNA region is more sensitive to nuclease degradation, suggesting higher exposure of DNA to nucleases between the mono-nucleosome and the chromatosome. Our data also highlight the predominance of double-stranded DNA of 121, 133, and 144 bp length resulting from double-strand breaks (Figure 1, A and B, and Table 3). Table 3 sums up the main causes of fragmentation and the resulting structures of cfDNA on the chromatin-derived particles, according to our WGS data.

Using WGS, we observed only 2 or 3 cfDNA size fragment populations; these corresponded to mono- or di-nucleosomes and traces of tri-nucleosomes, with the di-nucleosome–associated cfDNAs representing only a minor fraction of the total fragments. This confirmed the findings of a number of other studies that used either WGS or microcapillary electrophoresis (7, 38–44), performed in optimal preanalytical conditions (45). Note, some of those studies also revealed cfDNA of high molecular weight (2000 to 10,000 bp) at 10% to 20% (39, 41). When combining SSP-S and Q-PCR data concerning the 7 healthy individuals with the DII data concerning the 109 healthy individuals, we estimated the proportion of cfDNA inserted in mono-nucleosomes, di-nucleosomes, and chromatin of higher molecular size (>1000 bp) can be estimated as ranging 67.5% to 80.0%, 9.4% to 11.5%, and approximately 8.5% to 21.0%, respectively. Note, as indicated in Methods, these values are only indicative because of the inherent variation of cfDNA concentration as quantified when targeting a 320 bp amplicon.

These values correlated with the Chan et al. (23) data (15%–25%, and 10% in size fractions higher than approximately 300 bp and higher than ~500 bp, respectively) and with the values reported in several other studies (41). Bronkhorst et al. demonstrated that the 143B cancer cell line actively releases 2000 to 3000 bp sized segments of heterochromatin (46) and suggested that this secretion into the extracellular environment can induce a wide range of detrimental biological effects. Nevertheless, experiments with hemolytic plasma samples or matching serum, or using cell preservative tubes (44) or longtime storage (44), have highlighted the contamination of cfDNA samples with white blood cell DNA in the 300 to 450 bp and the 2000 to 11,000 bp size ranges, as reported in several studies (5, 39, 40, 47). It is difficult to specifically distinguish cfDNA from contaminating DNA using current techniques. Numerous conclusions in the literature regarding fragment size distribution are biased by obvious hematopoietic cell–DNA contamination, caused by improper preanalytical conditions (13, 48–51). For instance, Li et al. (48) observed a high proportion of high–molecular weight DNA in normal individuals and proposed an erroneous conclusion regarding its cfDNA distribution; this in turn misled the noninvasive prenatal test (NIPT) field into incorrectly postulating that greater NIPT performance is obtained by cfDNA size separation using a cutoff point of 300 or 500 bp, whereas most cfDNA clearly displays sizes below 300 bp. In contrast, our work (which was performed under optimal stringent preanalytical conditions, ref. 45) indicates that only a minor fraction of cfDNA is larger than that existing in mono-nucleosomes or transcription factor complexes circulating in the blood of healthy individuals. This suggests that the cfDNA detectable in plasma is present predominantly within those structures. Consequently, our data can be seen as supporting the notion that cfDNA sizing quality control must be performed to overcome biased conclusions regarding cfDNA size profiles, and to better analyze cfDNA, particularly in the case of a rare fraction of a specific cfDNA population (i.e., mutant cfDNA in oncology or fetal cfDNA in NIPT). For instance, we postulate that the cfDNA extract of healthy individuals displaying a fraction of di-nucleosome–associated DNA fragments over 20% should only be taken into consideration with considerable reserve. Fragmentation should therefore be considered as a parameter that must be monitored in order to ensure quality control (11, 45).

### Comparing cfDNA size profiles from healthy and cancer individuals

Because cancer is one of the most researched pathological conditions in the cfDNA field, our study sought to determine if fragmentation could provide a different perspective on the structure of cfDNA derived from cancer patients, as compared with that deriving from individuals of normal physiological condition, as described above. This exploratory study was based on the blinded examination of 7 plasma samples from healthy individuals and of plasma from 7 mCRC patients presenting a wide variation in MAF (0.9%, 3.2%, 14.4%, 23.3%, 47.3%, 54.7%, and 68.6%). Thus, it was possible to study the cancer cfDNA size profile across a wide range of malignant (mutant) cell-derived cfDNA. The cfDNA in cancer patients derives from the malignant cells, the tumor microenvironment cells (endothelial, stromal, immunological/lymphocytic cells), or the germinal cells. We and others have previously demonstrated that mutant cfDNA frequency varies widely in the plasma of cancer patients, independent of the stage of the disease and tumor size (19, 20). Nevertheless, we assume that the plasma DNA of mCRC patients exhibiting a high MAF (68.6%) displays characteristics very similar to cfDNA deriving from cancer/malignant cells (Table 4 and Table 5).

Overall, the plasma cfDNA of the cancer patients showed similar size profiles to those of healthy subjects and revealed the footprint of chromatin structures, in both DSP-S and SSP-S analysis. Our WGS study, however, clearly highlights differences in the plasma cfDNA fragment size range below 1000 bp, between cancer and healthy subjects: (a) cancer patients have more cfDNA fragments under 166 bp and less from 166 to 250 bp; (b) a size curve shoulder at 145 bp appears more pronounced in cancer individuals; and (c) these differences correlated directly with the proportion of tumor mutant (malignant) cfDNA.

As previously observed by Jiang et al. (22) in hepatocarcinoma cancer patients, the size profile obtained from conventional DSP-S showed a subtle but reliable difference between cancer and healthy subject–derived cfDNA. In our study, while DSP-S revealed a monomodal population of cfDNA peaking at 166 to 167 bp in both subject groups, we observed a moderate increase (10%–20%) of fragments between 90 and 166 bp, and a moderate decrease (<10%) between 166 and 250 bp in cancer patients, as compared with healthy individuals. As there is little or no variation in cfDNA size profiles among healthy subjects, as observed here and elsewhere (52–54), even subtle but reliable differences in size profile in the cancer cfDNA fragment population are potentially significant. Using either DSP-S or SSP-S analysis, the determination of the difference of cumulative frequencies demonstrated that, for cancer patient–derived cfDNA, the increase in fragment numbers was optimal around 160 bp. This indicates that cfDNA from tumor cells is more fragmented than that from healthy individuals. Note, the size profile of the cfDNA of the mCRC patient with the lowest MAF (0.9%) was not significantly different from that of the healthy individuals. Indeed, the fact that these differences increased with MAF tends to validate our observation of the differences between cancer and healthy cfDNA. Accordingly, the curve shoulder appearing at 145 to 155 bp in cancer patients would appear to be reliable. It will be remembered that 145 bp corresponds to DNA wrapped around a nucleosomal core unit (167 bp) minus a linker fragment of approximately 20 bp. We hypothesize that particles containing 145 and 166 bp DNA fragments are more stable than ones containing 153 bp fragments, due to the high nuclease sensitivity of the approximately 20 bp linker fragment. Consequently, our data showed that there are more cfDNA fragments in chromatosomes than in mono-nucleosomes, in healthy as compared with cancer subjects (Table 6 and Supplemental Table 2A). This in turn leads us to postulate that tumors have elevated or different DNase activity as recently postulated (7, 55).

The mean proportion of cfDNA over 320 bp in 104 all-stage CRC patients was estimated as approximately 0.4% by the *KRAS* intron 3 Q-PCR system. Because of the variation observed in the size profile of CRC patient–derived cfDNA relative to their MAF, the percentage range of the cfDNA fragment size populations cannot be estimated when combining data obtained by DSP-S, SSP-S, and Q-PCR analysis. Taken as a whole, however, the data reveal that the greater the MAF, the greater the number of fragments below 320 bp, and the fewer the number of fragments over approximately 1000 bp. Although these values are only indicative (Methods), they can be directly compared with those obtained in healthy individual plasma. As a consequence, in addition to the subtle difference in size profile within the 30 to 250 bp (nt) range, as previously observed in our study, the presence of a significant fraction (~8.5%–21%) of cfDNA with a fragment size over 1000 bp appears to be a landmark of healthy individual plasma (as compared with cancer patients), so long as the plasma cfDNA extracts are free of contaminating blood cell DNA. This finding confirmed the observation we previously made in xenograft mouse models and human plasma, that cfDNA from cancer patients is more fragmented than that of healthy individuals, when also considering fragment sizes over ~300 bp (16, 18, 29, 32). This has been convincingly established in the field, using various analytical methods (10, 18, 22, 56).

### CfDNA fragmentation analysis or “fragmentomics” as a cognitive or diagnostic tool

#### Toward a cancer screening test.

In addition to previously providing a proof-of-principle approach in using specific size fractions, size ratios, or size fraction ratios from cfDNA fragment size profile to distinguish cancer and healthy individual plasma (16, 29), our in-depth scrutiny of WGS size profiles offered another clear-cut assessment method for making such a distinction (Table 6 and ref. 57). Our initial observations (16, 29, 57) and the data presented here were confirmed using the Delfi cancer screening approach (28). The determination and evaluation of an algorithm combining different fragmentomics parameters is currently underway in our laboratory. Moreover, one of our recent reports (58) includes fragmentation indexes in a panel combined with other biomarkers, as a means of evaluating a machine-learning-assisted cancer screening test.

#### Diagnostics in oncology.

We first demonstrated that cfDNA fragments less than 100 bp were more frequent in cancer patients than in healthy subjects (6, 9, 29) and that the size of mutant cfDNA fragments whose sequence contained a mutation is shorter than that of the corresponding WT sequence (17). This observation has been clearly confirmed by Jiang et al. (22) and recently by Garlan et al. (59). Snyder et al. (8) pointed out the value of examining cfDNA fragmentation as a means of determining their tissue of origin and thus providing potential clues as to individual physiological states as a diagnostic aid, particularly in cases of cancer. Selection of fragments between 90 to 150 bp, using targeted and whole-genome sequencing approaches, could enrich the tumor DNA up to 11-fold (26). Hence, isolation of short cfDNA fragments appears as a means of enriching tumor variants and improving the correction of PCR- and sequencing-associated errors, especially in theragnostic testing (60).

#### Fragmentomics in other clinical fields.

Several reports have shown a clear, subtle, and reliable difference in size profile below 300 bp between fetal and maternal cfDNA (26). A parallel can be drawn between these cfDNA size profile differences and those that exist between cancer cfDNA and the cfDNA of healthy subjects. Remarkably, increases of fragment size within the 80 to 166 bp range and moderate decreases within the 166 to 220 bp range have also been observed.

CpG methylation, which is linked to an open chromatin structure and thus may be more accessible to native endonucleases (61), as well as difference of DNase activity and DNase species (7, 55), may contribute to the observed size difference. It is likely that some other physiological conditions may stimulate cells to produce cfDNA, and thus alter its size profile, i.e., lymphocytic cells during or after intense effort, or the immune cells after organ transplant.

#### CfDNA tissue of origin.

We unveiled here differences in the intimate cfDNA size profile at nucleotide level, allowing the characterization of the malignant or healthy cell origin of cfDNA extracts from blood samples. By generating maps of genome-wide in vivo nucleosome occupancy, Snyder et al. (8) and Lehmann et al. (36) revealed that cfDNA harbors footprints of transcription factors and that the origin of cfDNA tissue or cell type can be inferred from the correlation of nucleosome spacing. These 2 pivotal works extend considerably the scope of fragmentomics so that it could now encompass noninvasive monitoring of numerous diseases and of normal physiological conditions.

### Limitations and future directions

Our study has several limitations. Although we established the presence of cfDNA longer than 1000 bp in healthy individual plasma, we could not characterize the structure of this cfDNA population any further. Specific methods to do so are as yet unavailable. Furthermore, while sequencing analysis of the plasma of the 7 healthy individuals gave nearly identical size profiles, the number of plasma samples used for studying sizes below approximately 1000 bp is too low to consider our study anything more than exploratory. Confirmation performed on a large cohort remains necessary to demonstrate that all the discriminating factors revealed here have potential application in a screening test, as was convincingly but partially demonstrated by Cristiano et al. (28). Also, the WGS study on cfDNA from cancer patients was derived exclusively from mCRC patients. In addition, this study does not take into consideration mitochondria-derived cfDNA; similar investigation, therefore, should be performed that takes into account the growing interest of the clinical potential of mitochondrial cfDNA analysis (11). Finally, the different commercial DNA extraction kits were found not all equally efficient at extracting DNA of specific sizes (45). This study used a single method to prepare cfDNA; while that method was validated under a stringent preanalytical guideline (45), we nonetheless further confirmed our data using a capillary electromobility assay, as well as the conventional phenol/chloroform extraction method (Supplemental Figure 9).

It has confirmed our earlier hypothesis that size profiling, or fragmentomics (62), is a valuable strategy for characterizing cancer individuals (ref. 16, Table 6, and Table 7); as such, it offers a possible alternative or synergistic supplement to the strategy of searching for cancer-associated mutations — a strategy that, it must be noted, has recently shown false positivity (63). For these reasons, we are convinced that specific cfDNA structures, as observed by fragmentomics (6, 10, 28, 34), methylation (64, 65) or nucleosome positioning (8, 35), possess significant potential to improve diagnostics and early cancer detection.

## Methods

### Clinical samples.

The blood samples of healthy individuals (*n* = 7) were obtained from the Etablissement Français du sang (EFS, Supplemental Table 4). Blood samples from stage IV CRC patients (*n* = 7; Supplemental Table 4) were collected at the Montpellier Cancer Institute (Val d’Aurelle) and from the SIRIC Montpellier network. All individuals signed an informed consent form. Samples were handled according to a preanalytical guideline previously established by our group (45). In order to calculate a fragmentation index, a DII was generated in an ad hoc study using 109 control healthy subjects, sourced from the EFS, and 104 CRC patients of various stages (Supplemental Table 4), sourced via the SIRIC Montpellier network.

### Plasma isolation and cfDNA extraction.

All blood samples were collected in 4-milliliter EDTA tubes. The blood was then centrifuged at 1200*g* at 4°C for 10 minutes. The supernatants were isolated in sterile 1.55 mL Eppendorf tubes and centrifuged at 16,000*g* at 4°C for 10 minutes. Afterward, the plasma was either immediately used for DNA extraction or stored at –20°C. CfDNA was extracted from 1 mL of plasma using the QIAmp DNA Mini Blood kit (Qiagen) according to the “Blood and body fluid protocol.” DNA extracts were kept at –20°C until used. The preanalytical conditions we followed are described (45).

### Preparation of sequencing libraries and size profile analysis by deep sequencing.

Preparation of sequencing libraries as well as WGS are detailed in Supplemental Methods, Appendix 1. Note, the lower and upper size limits of detection by sequencing carried out under these conditions are estimated to be 20 to 30 bp and approximately 1000 bp, respectively.

### Fractional size distribution by Q-PCR.

Fractional size distribution by Q-PCR was performed as previously described (6, 9, 18, 29). Specific Q-PCR systems, calculation, presentation of the results, and limitations of this study are detailed in Supplemental Methods, Appendix 2, and Supplemental Figure 10.

### Determination of the cfDNA MAF.

MAF corresponds to the proportion of cfDNA fragments within a plasma extract bearing a targeted mutation. MAF was determined using the IntPlex assay, which is clinically validated (19), by testing 28 mutations on *KRAS*, *BRAF*, and *NRAS* genes actionable in mCRC management care (20) (Supplemental Methods, Appendix 3).

### Statistics.

Statistical analysis was performed using the GraphPad Prism V6.01 software. Where appropriate data were log transformed prior to statistical analysis. The Student’s *t* test, 1 tailed, was used to compare means. A *P* value of less than 0.05 was considered statistically significant; *P < 0.05, **P < 0.01; ***P < 0.001; ****P < 0.0001.

### Study approval.

We included mCRC patients from the screening procedure of the ongoing UCGI 28 PANIRINOX study (NCT02980510/EudraCT 2016-001490-33). Written informed consent was requested. Healthy individual blood samples were obtained from the EFS.

## Author contributions

ART designed the study, developed the methodology, analyzed the data, and prepared the manuscript. CS, ZAAD, BP, EP, and RT performed the experiments. CS and BR prepared the manuscript. BR, TM, PB, CS, ZAAD, BP, EP, RT, and ART discussed the results and approved the manuscript.

## Supplementary Material

Supplemental data

## Figures and Tables

**Table 1:**
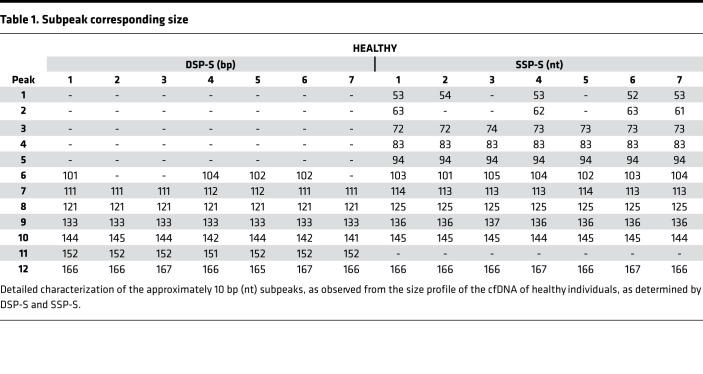
Subpeak corresponding size

**Table 2:**
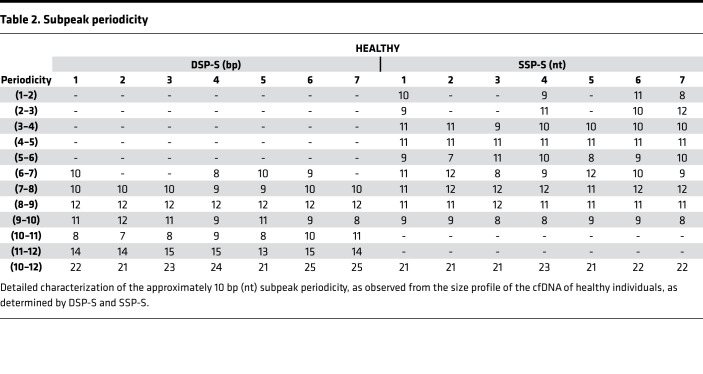
Subpeak periodicity

**Table 3:**
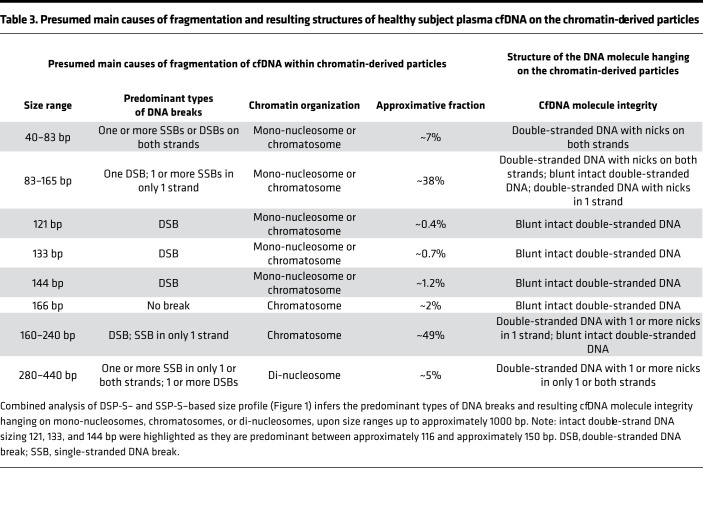
Presumed main causes of fragmentation and resulting structures of healthy subject plasma cfDNA on the chromatin-derived particles

**Table 4:**
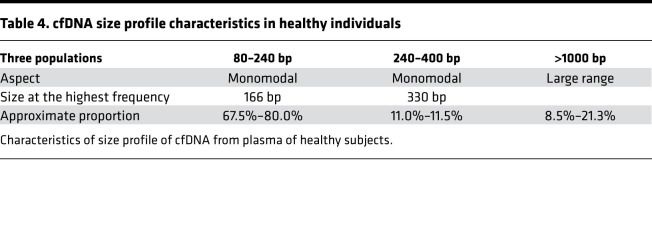
cfDNA size profile characteristics in healthy individuals

**Table 5:**
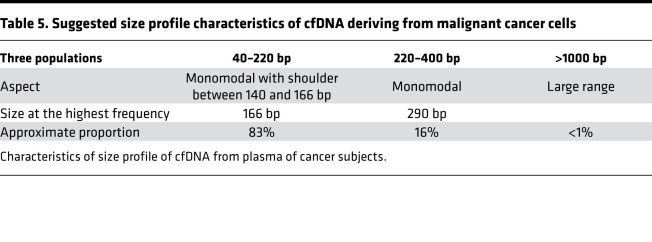
Suggested size profile characteristics of cfDNA deriving from malignant cancer cells

**Table 6:**
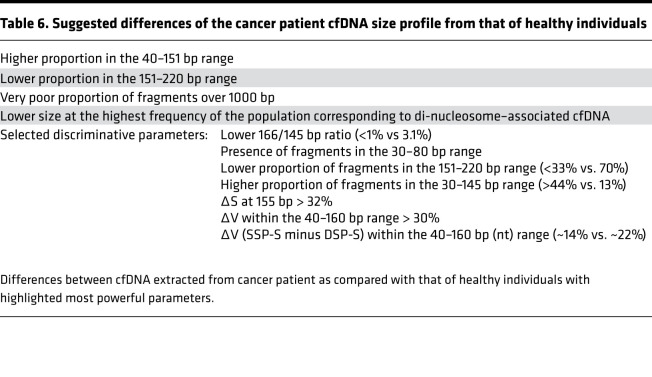
Suggested differences of the cancer patient cfDNA size profile from that of healthy individuals

**Table 7:**
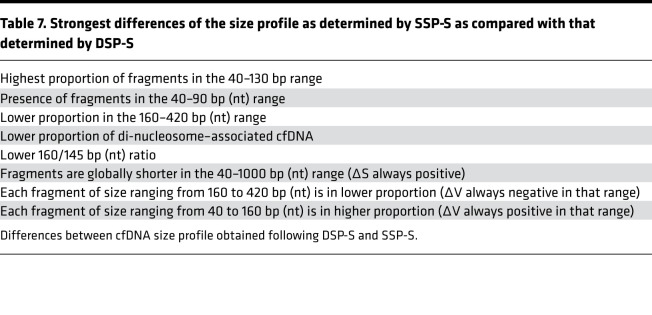
Strongest differences of the size profile as determined by SSP-S as compared with that determined by DSP-S

## Version 1 02/11/2021

In-Press Preview

## Version 2 04/08/2021

Electronic publication
