# Supplementary material for: Circulating nuclear DNA structural features, origins, and complete size profile revealed by fragmentomics
Source: JCI Insight. 2021 Apr 8;6(7):e144561. doi: 10.1172/jci.insight.144561 (PMC8119211; doi:10.1172/jci.insight.144561)
Supplement: Supplemental data [file jciinsight-6-144561-s144.pdf]

Supplemental data

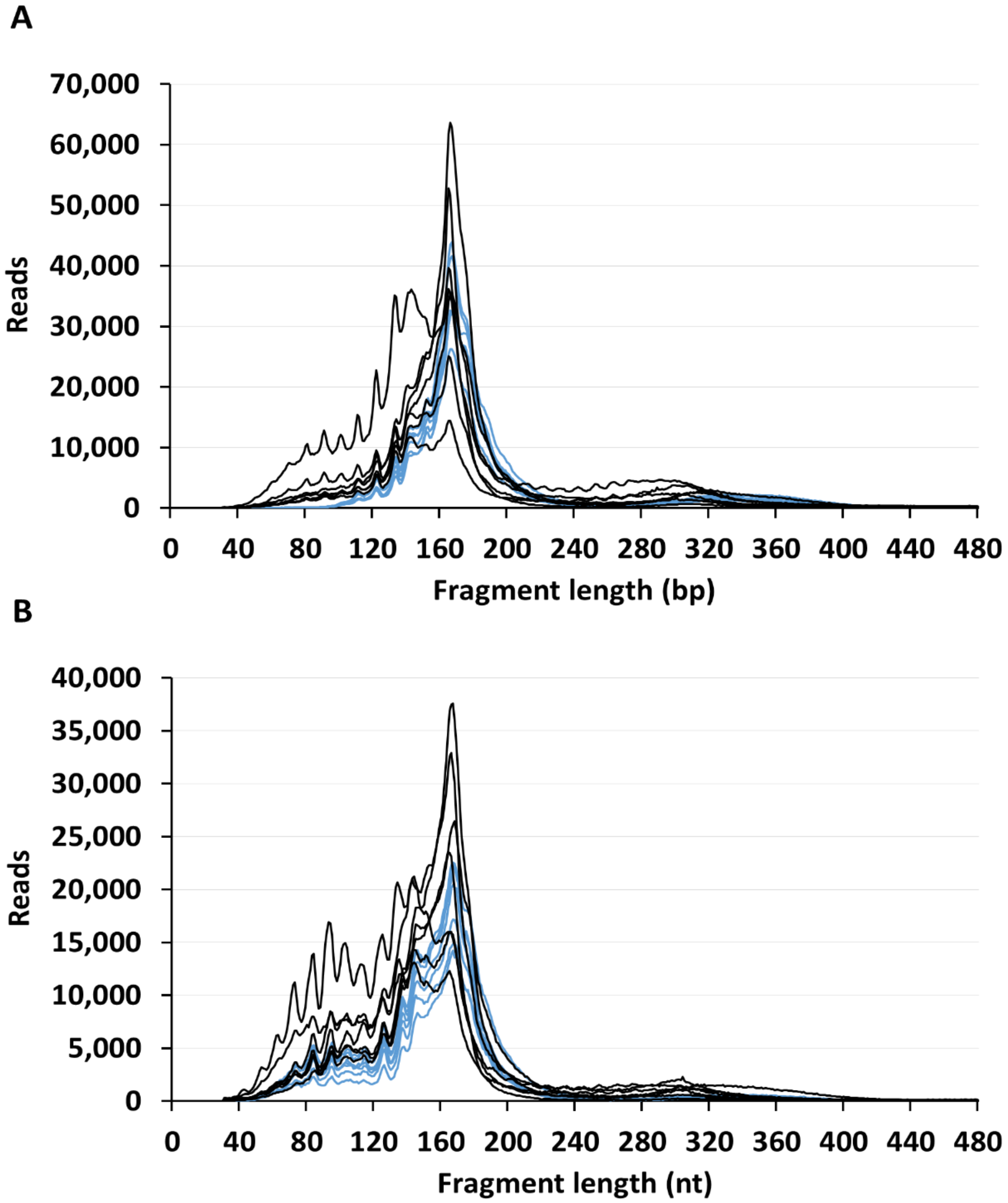

Supplemental Figure 1: Value of reads as determined by DSP-S (A) and SSP-S (B) of the seven healthy individuals (blue lines) and the seven cancer patients (black lines). Fragment length is expressed either as base pair (bp) or nucleotides (nt) when analyzing DSP-S or SSP-S data, respectively.

Box plots DII cancer patients vs healthy individuals

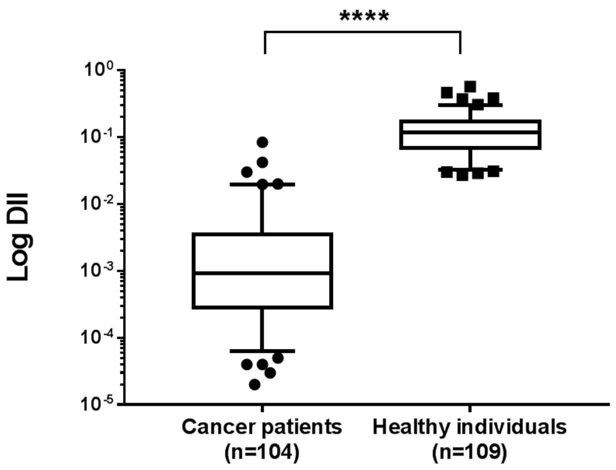

	Cancer patients	Healthy individuals
Number of values	104	109
Minimum	2.00E-05	0.027
25% Percentile	0.000285	0.069
Median	0.00091	0.119
75% Percentile	0.003475	0.169
Maximum	0.084	0.565
5% Percentile	6.25E-05	0.032
95% Percentile	0.01945	0.2975
Mean	0.004193	0.1344
Std. Deviation	0.0102	0.09133
Std. Error of Mean	0.001	0.008747
Lower 95% CI of mean	0.002209	0.117
Upper 95% CI of mean	0.006178	0.1517
Sum	0.4361	14.65

Supplemental Figure 2: Comparison of the DNA Integrity index (DII) value in cancer patients versus healthy subjects. Box plot analysis (left) and statistics (right).

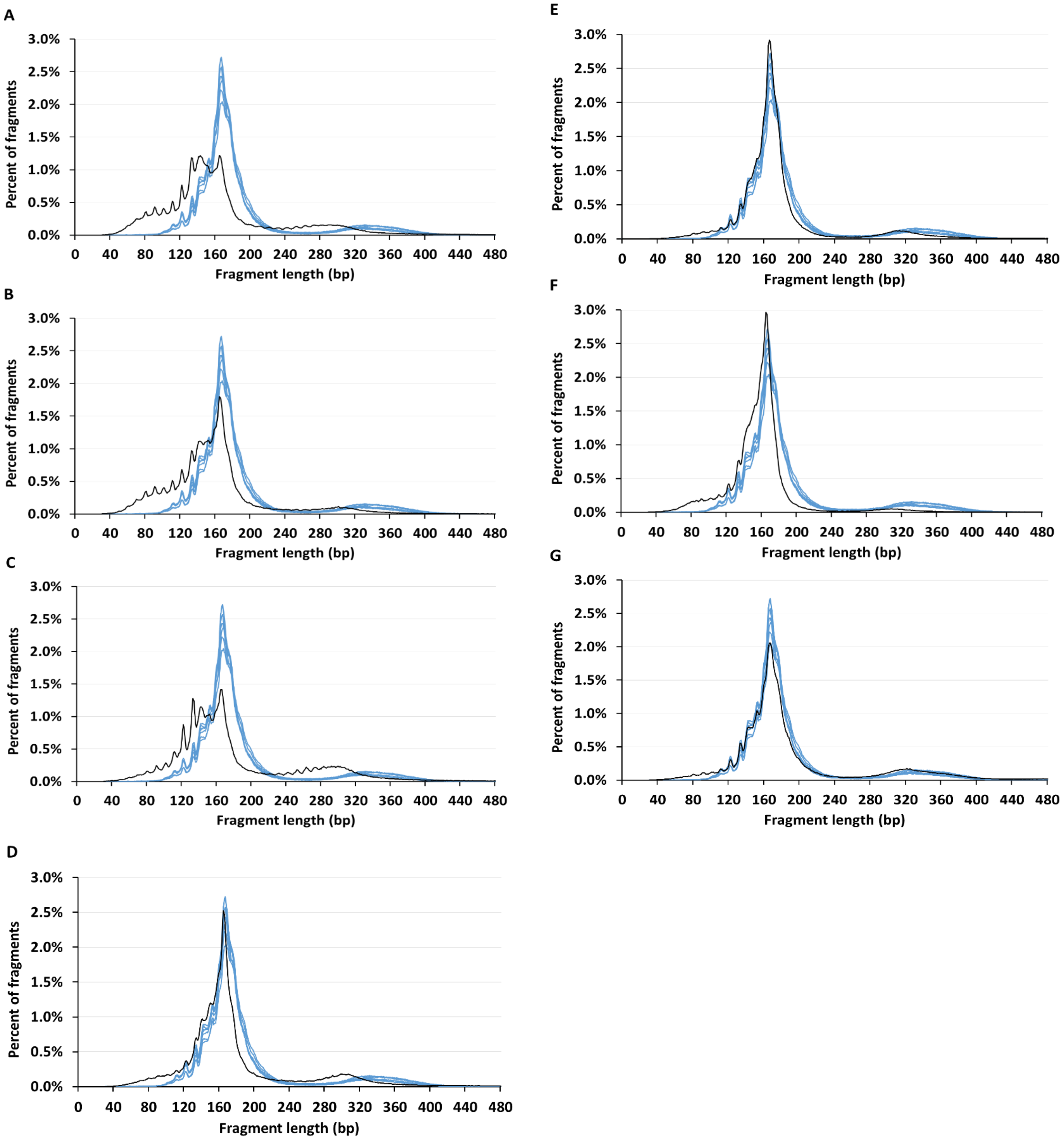

Supplemental Figure 3: Comparison of the cfDNA size profile (as determined by DSP-S) of the cfDNA extracted from seven mCRC patient plasma (black line) (A,B,C,D,E,F and G, respectively) with cfDNA size profiles of the seven healthy individuals (blue lines); A,B,C,D,E,F and G, (68.5%, 54.6%; 47.3%, 23.3%, 14.3%, 3.2%, and 0.9% total MAF, respectively); bp, base pairs.

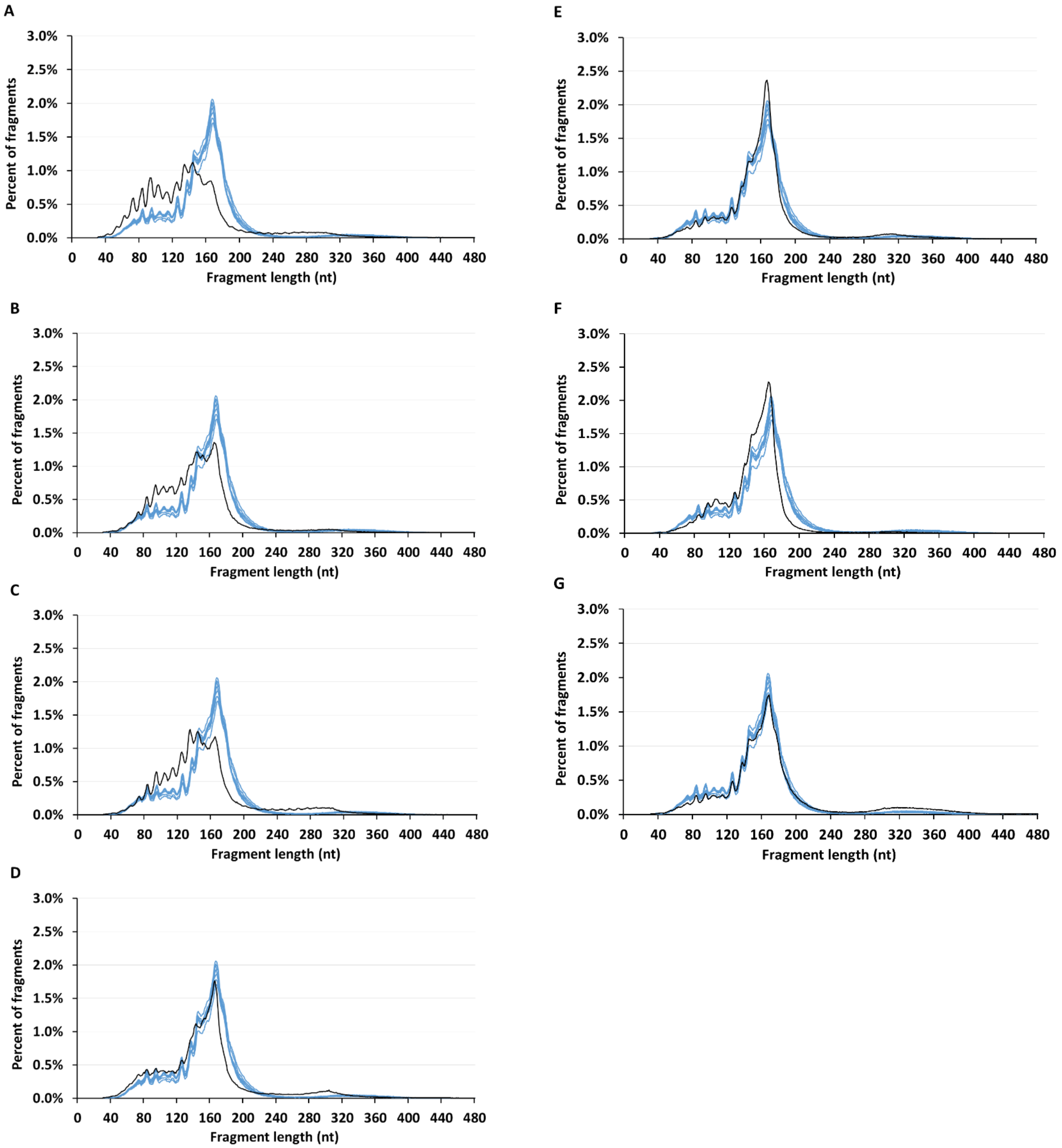

Supplemental Figure 4: Comparison of the cfDNA size profile (as determined by SSP-S) of the cfDNA extracted from seven mCRC patient plasma (A,B,C,D,E,F and G, respectively) with cfDNA size profiles (black line) of the seven healthy individuals (blue lines); A,B,C,D,E,F and G, (68.5%, 54.6%; 47.3%, 23.3%, 14.3%, 3.2%, and 0.9% total MAF, respectively); nt, nucleotides.

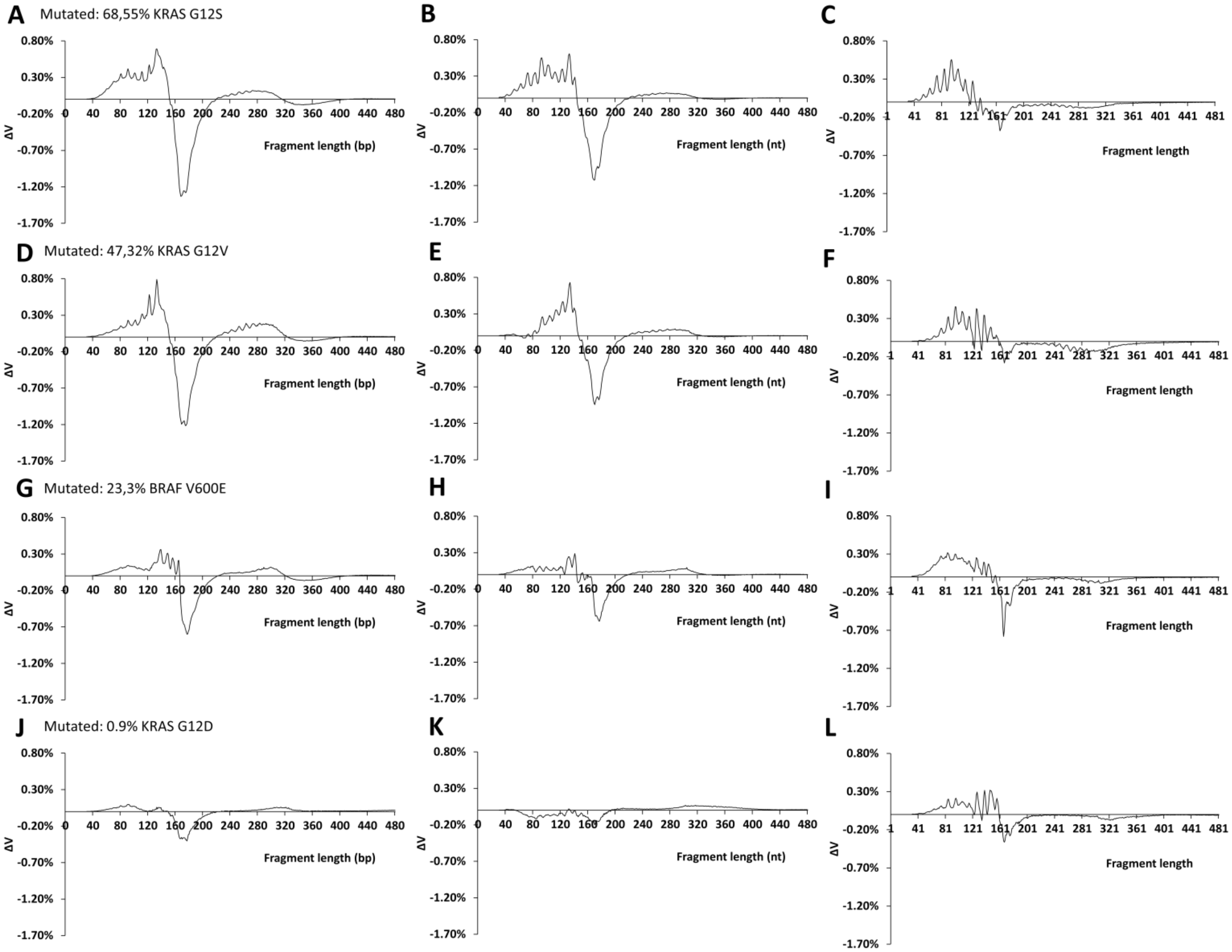

Supplemental Figure 5: Difference in frequency (△V) curves of four illustrative cancer patients, as determined by DSP-S (A, D, G, and J), SSP-S (B, E, H, and K), and subtracting DSP-S derived values from SSP-S derived values (C, F, I, and L). A, B, and C (MAF, 68.6%); D, E, and F (MAF, 47.3%); G, H, and I (MAF, 23.3%); and J, K, and L (MAF, 0.9%).

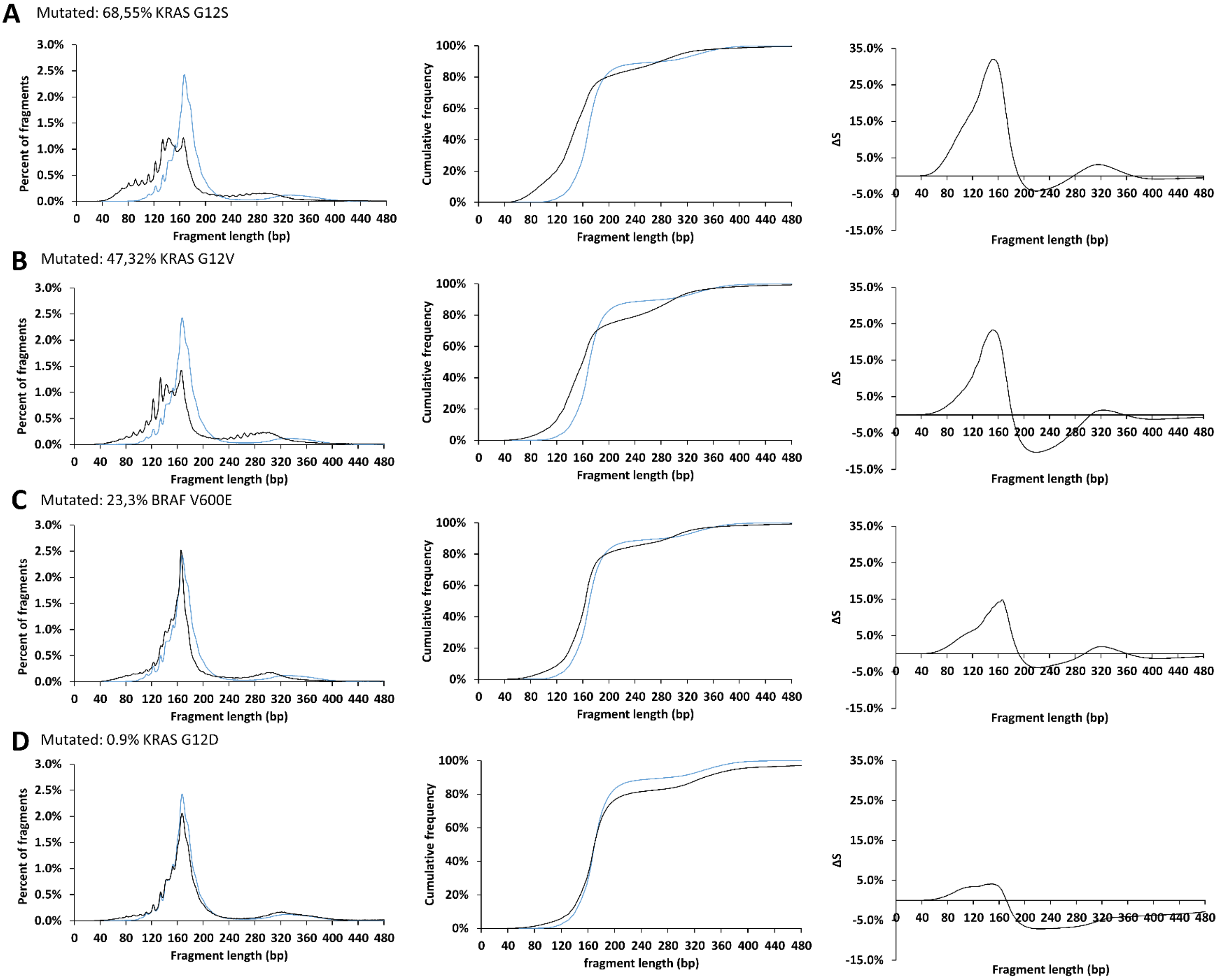

Supplemental Figure 6: Comparison of the cfDNA size profiles of the four illustrative cancer patients (black line) and the mean size profile of the seven healthy individuals (blue line), as determined by DSP-S (left panel); curves of the cumulative frequencies (middle panel); and the difference in cumulative frequencies, denoted as ΔS, between cancer minus healthy (right panel). bp, base pairs. A, B, C, and D (68.6%, 47.3%, 23.3%, and 0.9% total MAF, respectively).

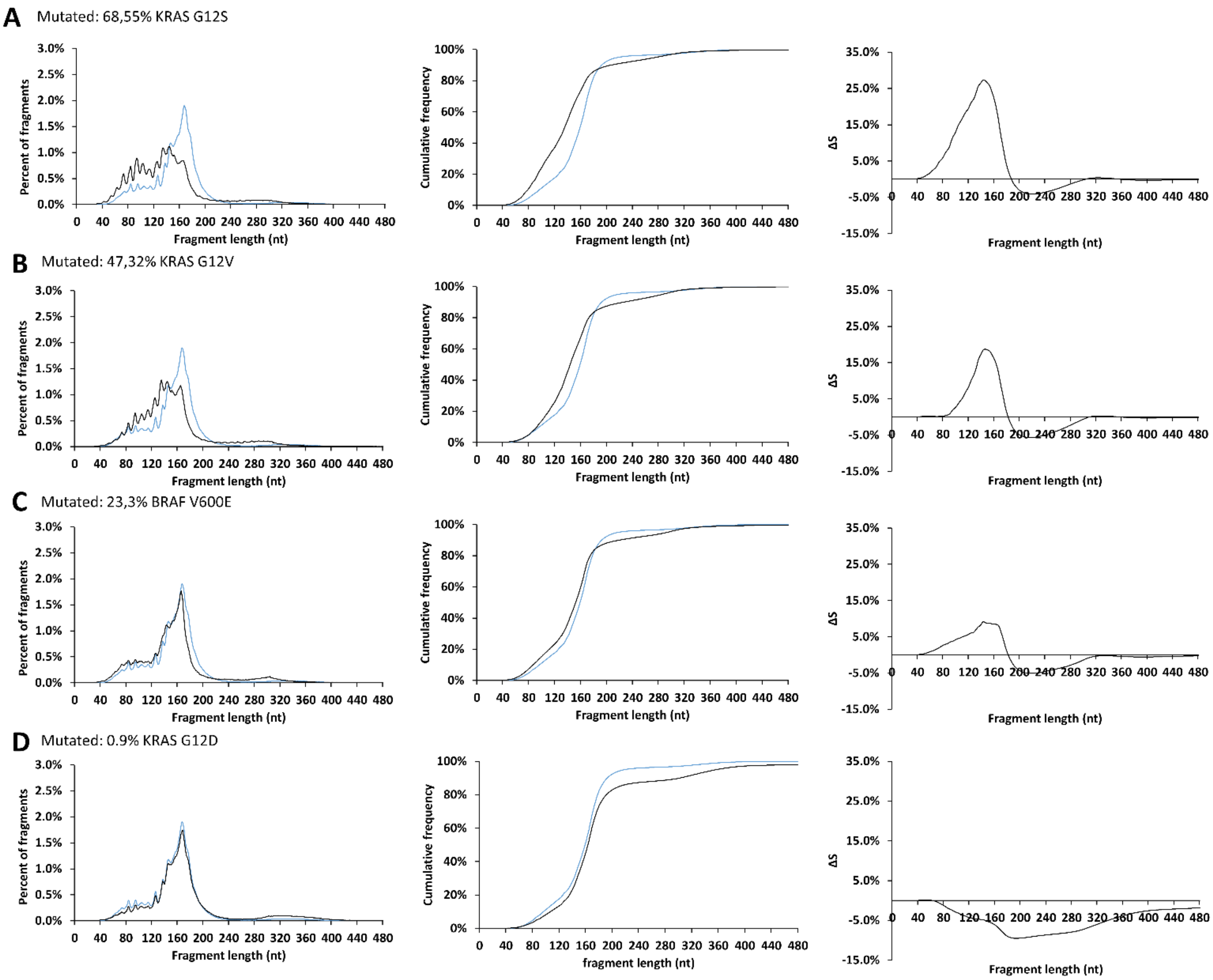

Supplemental Figure 7: Comparison of the cfDNA size profiles of the four illustrative cancer patients (black line) and the mean size profile of the seven healthy individuals (blue line), as determined by SSP-S (left panel); curves of the cumulative frequencies (middle panel); and the difference in cumulative frequencies, denoted as ∆S, between cancer minus healthy (right panel). nt, nucleotides. A, B, C, and D (68.6%, 47.3%, 23.3%, and 0.9% total MAF, respectively).

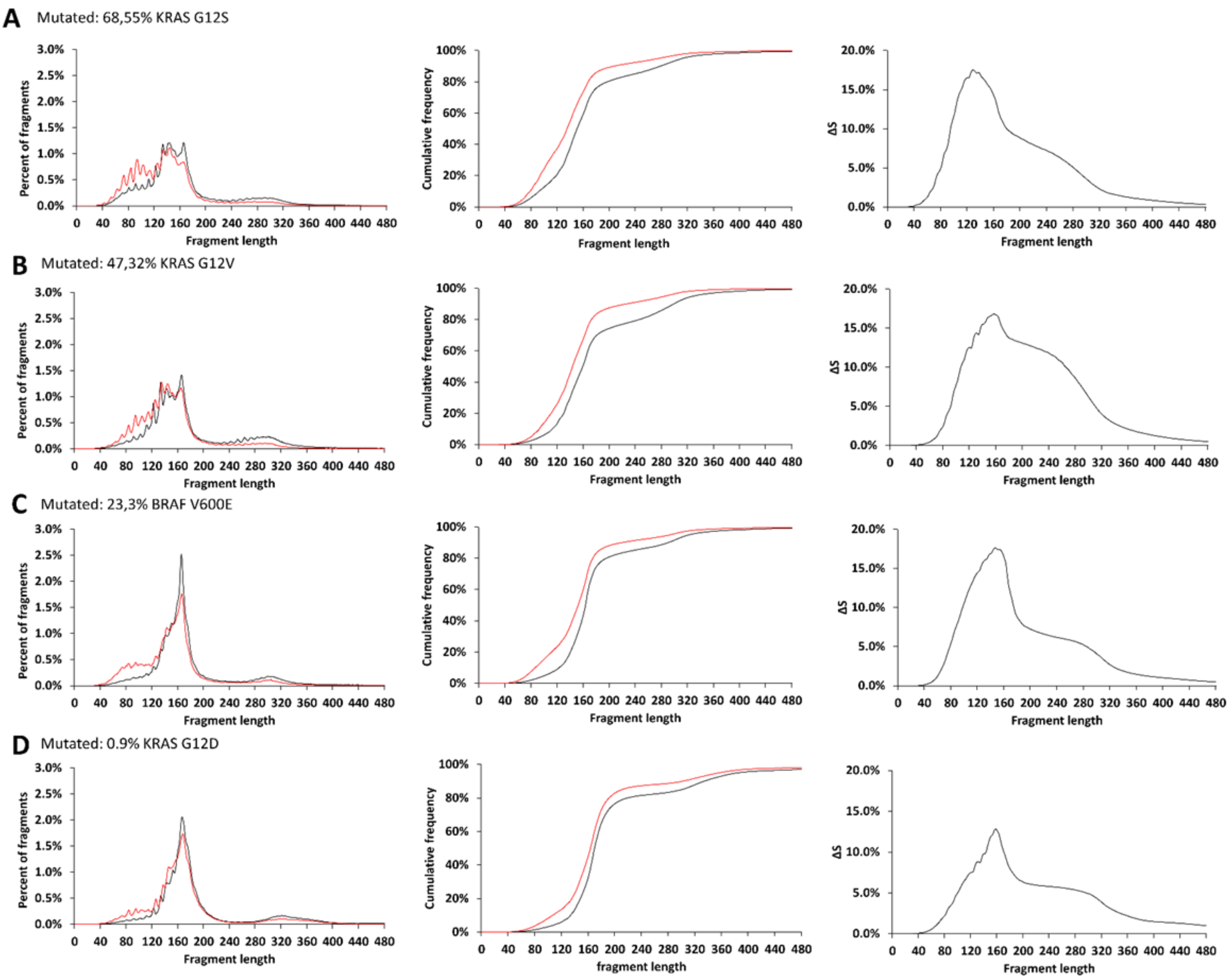

Supplemental Figure 8: Comparison of the cfDNA size profiles obtained from four illustrative cancer patients, as determined by DSP-S (black line) and by SSP-S (red line); curves of the cumulative frequencies of SSP-S and DSP-S values (middle panel); and the difference in cumulative frequencies, denoted as ΔS, between SSP-S minus DSP-S (right panel). Fragment length (bp/nt). A, B, C, and D (68.6%, 47.3%, 23.3%, and 0.9% total MAF, respectively).

A

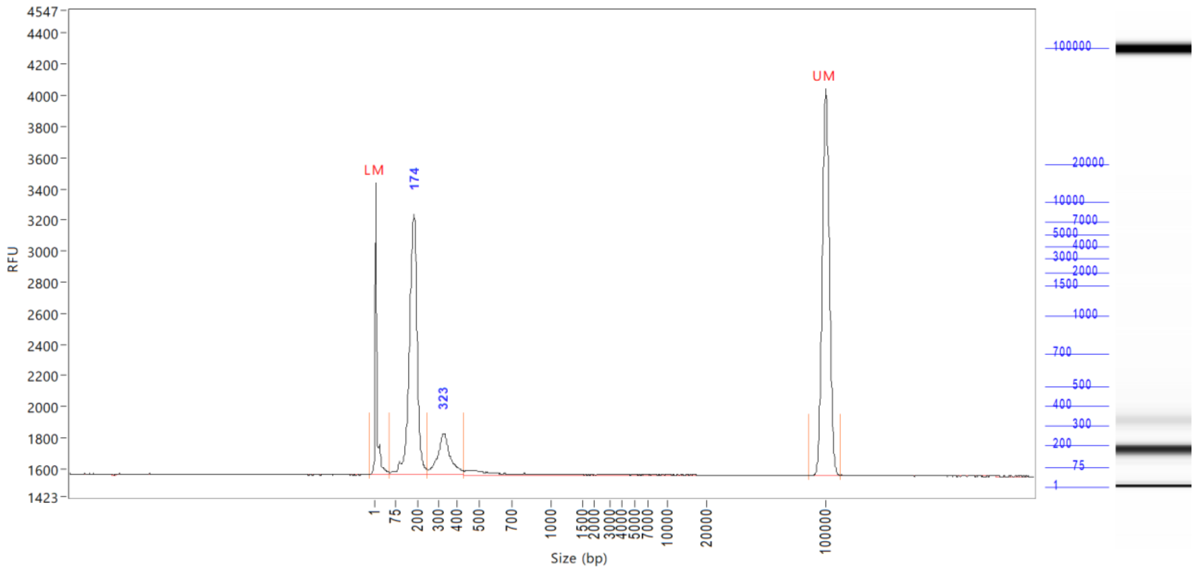

B

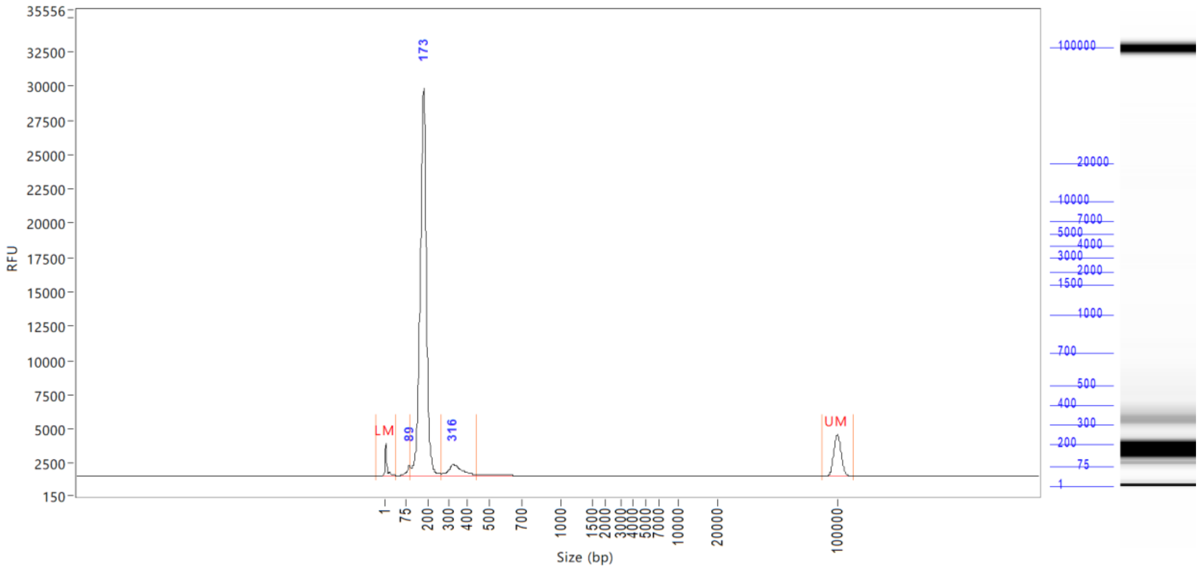

	Healthy individual				CRC patient			
	Qiagen		Ph/Chl		Qiagen		Ph/Chl	
Range (bp)	% of Total	Avg. Size (bp)	% of Total	Avg. Size (bp)	% of Total	Avg. Size (bp)	% of Total	Avg. Size (bp)
80-230	51.8	151.0	66.0	159.0	78.8	166.0	92.6	166.0
280-450	8.5	345.0	8.1	356.0	16.8	336.0	5.4	342.0
1300-10000	25.8	4702.0	14.1	5956.0	0.9	4276.0	0.3	4008.0
Total conc of extract by FA, ng/µl	0.05		0.02		0.8		7.0	
Conc in ng/ml plasma	1.5		0.5		39.4		349.1	

Supplemental Figure 9: Illustration of the size profile of the cfDNA of a mCRC patient extracted by Qiagen mini blood kit (A) and by the Ph/chl extraction method (B), as determined by capillary electrophoretic mobility assay. Proportion (%) of fragments in selected size ranges, as determined

following Qiagen and Ph/Chl extraction methods of cfDNA from illustrative healthy and cancer subjects (Bottom Table). CfDNA size profile may also be assessed by capillary electrophoretic mobility assay that offers advantages in terms of cost, simplicity, rapidity and size limit (up to 20,000 bp) but has the disadvantages of being much less accurate, specific and sensitive, in particular for short fragments and low DNA dose input. Consequently, it rather corresponds to an estimation of the cfDNA size profile. Nevertheless, this method enables to display the general composition of the cfDNA fragment population as observed with using WGS and Q-PCR. All the data described here are obtained from cfDNA purified by the same extraction kit that is based on a silica-based column. Several reports indicated variation of the extraction yield in respect to small DNA size when comparing various extraction kits (1). In order to estimate any bias due to the extraction method, we compared by electrophoretic mobility assay the size profile as obtained by silica-based column and by the old-fashion and conventional Phenol/chloroform extraction methods. Overall, the size profiles of cfDNA obtained from both extraction methods are similar in either among healthy or among cancer individuals showing only low but significant variation when comparing the proportion of the cfDNA fragment populations (inserted Table). Altogether, these data consolidated the observation we described in this study by combining DSP-S, SSP-S and Q-PCR analysis. Capillary electrophoretic mobility assay was performed with using the Fragment Analyzer (Agilent) and the Phenol/chloroform extraction with using the Sigma kit.

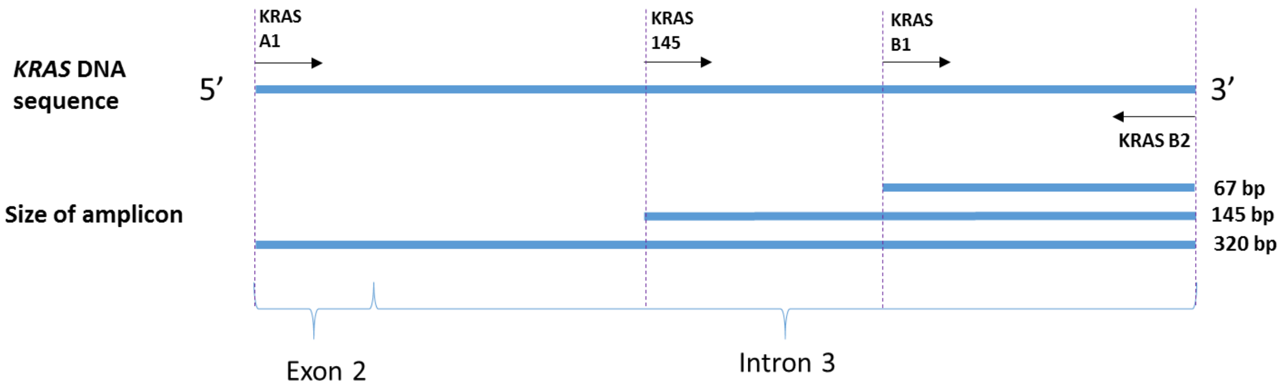

Sequence of primers

KRAS B2 (antisense) = 5' CCCTGACATACTCCCAAGGA 3'
KRAS B1 (sense) = 5' CCTTGGGTTTCAAGTTATATG 3'
KRAS 145 (sense) = 5' GATAAAGGTTTCTCTGACCA 3'
KRAS A1 (sense) = 5' GCCTGCTGAAAATGACTGA 3'

Fragmentation Index

DII = [cfDNA] fragments of 320 bp/ [cfDNA] fragments of 67 bp

Supplemental Figure 10: Scheme and sequences of the primer systems on the *KRAS* exon 2 gene.

A

	Dinucleosome associated fragment's % (250 to 400 bp)	
**ID patient**	**DSP**	**SSP**
**1**	8.0%	2.9%
**2**	9.1%	2.5%
**3**	12.9%	4.2%
**4**	10.0%	3.8%
**5**	12.9%	4.4%
**6**	12.8%	4.5%
**7**	8.0%	2.7%
**8**	12.5%	6.5%
**9**	7.7%	3.7%
**10**	17.9%	7.7%
**11**	12.3%	7.2%
**12**	7.6%	5.0%
**13**	3.2%	1.5%
**14**	13.8%	9.5%

B

	Dinucleosome associated peak	
**ID patient**	**DSP**	**SSP**
**1**	332	327
**2**	332	327
**3**	332	327
**4**	332	327
**5**	332	327
**6**	343	338
**7**	330	327
**8**	293	293
**9**	301	305
**10**	299	295
**11**	304	304
**12**	313	311
**13**	309	309
**14**	323	310

Supplemental Table 1: Proportion (%) of the 250 - 400 bp size fraction of cfDNA extracted from the plasma of the seven healthy (1 - 7) and the seven cancer subjects (8 - 14) as determined by DSP-S and SSP-S (A). Size at the di-nucleosome-associated peak (B).

A

Fragment size values (bp/nt)	HEALTHY (n=7)									CANCER (n=7)						
	% of fragments, DSP-S															
	Mean healthy	SD healthy	1	2	3	4	5	6	7	8	9	10	11	12	13	14
145	0.78%	0.00086	0.83%	0.77%	0.65%	0.84%	0.83%	0.69%	0.88%	1.18%	1.09%	1.07%	0.96%	0.89%	1.30%	0.79%
166	2.42%	0.00248	2.70%	2.67%	2.32%	2.43%	2.22%	2.02%	2.55%	1.20%	1.78%	1.41%	2.47%	2.92%	2.93%	2.05%
**166/145**	**3.08**	**0.32945**	**3.25**	**3.46**	**3.56**	**2.90**	**2.69**	**2.94**	**2.88**	**1.01**	**1.64**	**1.32**	**2.57**	**3.29**	**2.26**	**2.62**
30 to 220	87.28%	0.02809	90.40%	89.11%	84.51%	87.80%	84.60%	84.17%	90.36%	83.11%	89.52%	77.01%	83.47%	90.38%	96.00%	80.16%
220 to 440	12.72%	0.02797	9.61%	10.90%	15.50%	12.17%	15.35%	15.82%	9.66%	16.17%	10.08%	22.05%	15.42%	8.94%	3.82%	16.42%
**30 to 145**	**13.40%**	**0.01827**	**14.12%**	**11.62%**	**10.37%**	**14.50%**	**14.61%**	**13.05%**	**15.49%**	**44.05%**	**40.09%**	**35.81%**	**24.60%**	**17.35%**	**28.02%**	**17.47%**
80 to 166	41.47%	0.03638	44.31%	42.13%	36.40%	43.66%	41.55%	36.64%	45.61%	60.57%	62.02%	56.15%	54.40%	49.32%	66.32%	42.03%
	% of fragments, SSP-S															
145	1.18%	0.00095	1.19%	1.29%	1.12%	1.25%	1.16%	1.00%	1.22%	1.09%	1.21%	1.24%	1.08%	1.15%	1.49%	1.10%
166	1.86%	0.00152	1.99%	2.03%	1.96%	1.82%	1.74%	1.60%	1.88%	0.84%	1.35%	1.13%	1.76%	2.36%	2.23%	1.69%
**166/145**	**1.58**	**0.09773**	**1.67**	**1.58**	**1.74**	**1.46**	**1.50**	**1.60**	**1.55**	**0.78**	**1.11**	**0.91**	**1.64**	**2.05**	**1.50**	**1.53**
30 to 220	95.16%	0.01154	96.21%	96.56%	94.38%	94.79%	94.18%	93.74%	96.26%	91.04%	94.86%	89.49%	90.08%	93.47%	97.94%	86.12%
220 to 440	4.80%	0.01136	3.77%	3.43%	5.59%	5.16%	5.75%	6.21%	3.72%	8.71%	5.03%	10.30%	9.51%	6.15%	1.99%	11.73%
**30 to 145**	**33.08%**	**0.02380**	**34.44%**	**31.74%**	**28.64%**	**33.66%**	**34.49%**	**32.62%**	**35.93%**	**60.38%**	**52.70%**	**51.76%**	**42.13%**	**30.64%**	**39.84%**	**28.05%**
80 to 166	56.88%	0.02775	59.02%	58.65%	53.31%	58.13%	56.92%	52.68%	59.43%	68.39%	71.86%	69.63%	62.58%	59.69%	74.96%	51.08%

B

	Fragment frequency - cancer vs healthy													
	DSP-S (bp)							SSP-S (nt)						
	8	9	10	11	12	13	14	8	9	10	11	12	13	14
ΔS (155 bp/nt)	31.8%	28.3%	23.0%	13.3%	5.4%	20.1%	3.9%	24.9%	19.2%	17.7%	8.7%	-1.9%	10.7%	-5.7%
ΔV (40 to 160 bp/nt)	29.96%	27.69%	21.41%	14.15%	6.40%	23.11%	3.32%	22.05%	18.06%	16.05%	8.46%	-0.79%	13.34%	-6.13%

C

	ΔV - SSP minus DSP													
Size range	1	2	3	4	5	6	7	8	9	10	11	12	13	14
40 to 80	4.82%	4.39%	4.46%	4.44%	5.01%	4.81%	5.09%	4.84%	0.79%	2.41%	5.36%	2.69%	0.94%	2.18%
40 to 120	15.80%	14.14%	13.03%	14.79%	15.91%	15.83%	16.43%	16.41%	9.81%	12.54%	14.36%	9.32%	8.57%	7.46%
**40 to 160**	**22.15%**	**23.26%**	**22.37%**	**21.13%**	**21.92%**	**21.94%**	**21.50%**	**14.13%**	**12.41%**	**16.68%**	**16.35%**	**14.84%**	**12.27%**	**12.59%**
80 to 120	11.24%	10.00%	8.80%	10.60%	11.19%	11.31%	11.63%	11.78%	9.05%	10.27%	9.27%	6.74%	7.67%	5.37%
130 to 160	4.48%	7.01%	7.61%	4.42%	4.36%	4.48%	3.17%	-3.28%	1.02%	2.40%	0.59%	4.13%	2.26%	3.89%
Cancer minus healthy mean (range 40 to 160)								-7.91%	-9.63%	-5.36%	-5.69%	-7.20%	-9.77%	-9.45%

Supplemental Table 2: Frequency at selected size, size range, or size ratio from healthy individuals (n=7) and cancer patients (n=7) (A). Difference in cumulative frequency (ΔS) at 155 bp (nt) when comparing cancer to healthy subject plasma (B). The difference between SSP-S and DSP-S for selected size ranges, denoted as ΔV (C).

A

	Subpeak corresponding size													
	CANCER													
	DSP-S (bp)							SSP-S (nt)						
**Peak**	**8**	**9**	**10**	**11**	**12**	**13**	**14**	**8**	**9**	**10**	**11**	**12**	**13**	**14**
**1**	-	-	-	-	-	-	-	42	41	41	42	42	41	42
**2**	-	-	-	-	-	-	-	53	52	53	53	53	54	55
**3**	-	-	-	-	-	-	-	63	63	64	64	65	-	63
**4**	71	70	-	-	-	-	-	73	72	72	74	74	73	73
**5**	81	80	80	80	81	85	80	84	83	83	84	84	84	84
**6**	91	90	90	92	92	90	91	93	93	93	95	95	94	95
**7**	101	101	101	101	101	101	101	102	103	103	104	105	103	104
**8**	111	110	111	111	112	110	111	113	113	113	114	115	114	114
**9**	122	121	121	123	122	121	122	125	124	124	126	126	125	126
**10**	133	132	132	134	134	133	133	134	135	135	-	138	137	137
**11**	143	141	141	141	-	144	143	144	142	142	143	145	144	145
**12**	-	151	151	150	152	152	152	151	151	151	153	-	-	-
**13**	165	164	164	165	166	164	167	166	164	164	166	167	163	168

B

	Subpeak periodicity													
	CANCER													
	DSP-S (bp)							SSP-S (nt)						
**Periodicity**	**8**	**9**	**10**	**11**	**12**	**13**	**14**	**8**	**9**	**10**	**11**	**12**	**13**	**14**
**(1-2)**	-	-	-	-	-	-	-	11	11	12	11	11	13	13
**(2-3)**	-	-	-	-	-	-	-	10	11	11	11	12	-	8
**(3-4)**	-	-	-	-	-	-	-	10	9	8	10	9	-	10
**(4-5)**	10	10	-	-	-	-	-	11	11	11	10	10	11	11
**(5-6)**	10	10	10	12	11	5	11	9	10	10	11	11	10	11
**(6-7)**	10	11	11	9	9	11	10	9	10	10	9	10	9	9
**(7-8)**	10	9	10	10	11	9	10	11	10	10	10	10	11	10
**(8-9)**	11	11	10	12	10	11	11	12	11	11	12	11	11	12
**(9-10)**	11	11	11	11	12	12	11	9	11	11	-	12	12	11
**(10-11)**	10	9	9	7	-	11	10	10	7	7	-	7	7	8
**(11-12)**	-	10	10	9	-	8	9	7	9	9	10	-	-	-
**(12-13)**	-	13	13	15	14	12	15	15	13	13	13	-	-	-

Supplemental Table 3: Detailed characterization of the ~10 bp (nt) periodicity sub-peaks observed from size distribution of cfDNA of cancer patients, as determined by DSP-S and SSP-S. Value at the peak (A) and periodicity (B). bp, base pair; nt, nucleotides.

A

	Number of individuals	Age (years)			Gender		Concentration of total cfDNA (ng/ml plasma)		
		Mean	Median	Range	Male	Female	Mean	Median	Range
**Healthy individuals**									
Combining WGS and Q-PCR study	7	37.5	31.0	26-59	51.2%	48.2%	1.23	0.8	0.6-2.3
Ad hoc Q-PCR study	109	48.5	51.5	24-72	52.4%	47.5%			
**Cancer patients**									
Combining WGS and Q-PCR study	7	62.6	62	45-74	57.2%	42.8%	77.11	50.24	8.3-295.3
Ad hoc Q-PCR study	104	73.6	75.0	51-91	55.9%	44.1%			

B

Mutational status of the WGS studied cancer patients (n=7)			
Patient ID	Point mutation	Concentration of total cfDNA (ng/ml plasma)	Mutant allele frequency (MAF)
8	KRAS G12S	13,9	68,6
9	KRAS G12C	45,3	54,7
10	KRAS G12V	295,3	47,3
11	BRAF V600E	67,9	23,3
12	KRAS G12V	59,0	14,4
13	BRAF V600E	50,2	3,2
14	KRAS G12D	8,3	0,9

Supplemental Table 4: Characteristics of the healthy individuals, of the cancer patients, and of the respective plasma extracted cfDNA. Description of the Q-PCR systems used by the IntPlex assay for measuring the MAF of the cancer patient plasma used in this study. All primer sets enable the detection of amplicons of size <100 bp. The IntPlex assay was performed as described (2). Oligoblocker and NRAS primer sequences will be provided upon written request.

Supplemental Methods Appendix 1

Preparation of sequencing libraries

DSP and SSP libraries were prepared. SSP allows the integration of single- and double-strand DNA in the library. DSP libraries were prepared with the NEB Next® Ultra™ II kit. SSP libraries were prepared with the Swift ACCEL-NGS® 1S PLUS kit. For both preparations, a minimum of 1ng of cfDNA was engaged without fragmentation, and each kit providers' recommendations were followed. Those recommendations can be summarized as follows: for DSP (with the NEB kit), Illumina paired-end adaptor oligonucleotides were ligated on repaired A-tailed fragments, then purified by solid-phase reversible immobilization (SPRI) and enriched by 11 PCR cycles with UDI primers indexing, then SPRI purified again; for SSP (with the Swift kit), heat denaturation was performed first, to allow the conversion of all DNA into single strands; this protocol allows, simultaneously, the extension of single-stranded fragments and the attachment of adapters to the end of those fragments; Adaptase simultaneously links an adapter to and extends their 3' end. The complementary strand was then synthesized by a primer extension. After SPRI purification, a second adapter was ligated at the other end, purified by SPRI, and the product enriched by 11 cycles of PCR, then purified by SPRI again. For both types of preparation, the SPRI purification was adjusted to keep the small fragments around 70 bp of insert. Finally, the libraries to be sequenced were precisely quantified by Q-PCR, in order to load the appropriate DNA quantity to the Illumina sequencer, and to obtain a minimum of 1.5 million of clusters.

The frequency (%) at each fragment size was calculated from the sequencing reads to the total of reads obtained in the library. The base pairs (bp) or the nucleotides (nt) are used as the fragment size distribution length unit when using DSP-S or SSP-S, respectively. When data from both DSP-S and SSP-S are compared, size or size range is expressed as bp (nt).

Size profile analysis by deep sequencing

All libraries were sequenced using a MiSeq500 or NovaSeq (Illumina) as paired-end 100 reads. Image analysis and base calling was performed by Illumina Real Time Analysis, using default parameters. The individual barcoded paired-end reads (PE) were trimmed with Cut adapt v1.10 to remove the adapters and discard trimmed reads shorter than 20 bp. Trimmed FASTQ files were aligned to the human reference genome (GRCH38) using the MEM algorithm in the Burrows-Wheeler Aligner (BWA) v0.7.15. The insert sizes were then extracted from the aligned bam files with the TLEN column

for all pairs of reads with an insert size between 0 and 1000 bp. DNA libraries and sequencing were done by IntegraGen SA, Evry, France.

The frequency of each fragment size separated by one bp or nucleotide was calculated by the ratio of the number of reads at each fragment size to the total number of fragments from 30 to 1000 bp (nt). Since the size profile generated by DSP-S or SSP-S relies on the presence of double- or single-strand DNA, respectively, the fragment size unit used for DSP-S and SSP-S is bp and nt, respectively. Note, the fragment sizes are offset by 3 bp for the two methods, which is consistent with damaged or non-flush input molecules, whose true endpoints are more faithfully represented in single-stranded libraries

Cumulative frequency distribution analysis was performed based on the size profile distribution data. ΔS was calculated from the cumulative frequency distribution data as the difference between cancer patients and healthy individuals in either DSP-S or SSP-S, or as the difference between SSP-S and DSP-S in both. Using the percentage frequency distribution data, "ΔV" was calculated as the difference between cancer patients and healthy individuals (cancer minus healthy) using either DSP-S or SSP-S, or as the difference between SSP-S and DSP-S (SSP-S minus DSP-S). When the number of fragments detected by SSP-S are higher than the number detected by DSP-S at a specific cfDNA size, the ΔV value is positive. When the number of fragments detected by SSP-S is lower than the number detected by DSP-S at a specific cfDNA size, ΔV value is negative. Thus, the ΔV value precisely indicates the respective position of both DSP- and SSP-S size profile curves, and at which size the curves intersect (ΔV value = 0).

Interpretation of data from figure 1 E and F

First, we calculated the differences in the cumulating frequencies, denoted as △S, from the mean fragment size profile (Figure 1C, D and E); in doing so, we found a sharp increase from 45 bp (nt) up to 166 bp (nt), followed by a sharp decrease down to ~200 bp (nt), and a slower decrease down to 420 bp (nt). Second, we plotted the differences of the frequency at each bp (nt) between SSP-S and DSP-S (SSP-S minus DSP-S), denoted △V (Figure 1F). △V varies periodically: it is positive from 45 to 160 bp (nt), then negative up to 440 bp (nt); maximal values plateauing between 90 and 150 bp (nt). The comparison of the mean curves (Figure 1C) and △V data (Figure 1F) clearly showed that SSP-S revealed a higher number of fragments in the 45 - 160 bp (nt) range, and a lower number of fragments in ranges from 160 - 250 bp (nt) and, to a lesser extent, 280 - 440 bp (nt). △V values highly vary between 120 and 160 bp (nt), from maximal to ~null values (especially at 123, 134, 143 bp (nt)). In addition, the courses of the DSP-S and SSP-S derived values are nearly equivalent between 166 and 200 bp (nt), as

observed on the slope of their respective size profiles (Figure 1C), and the ΔS (Figure 1D and E) or ΔV (Figure 1F) curves.

Supplemental Methods Appendix 2

Fractional size distribution by Q-PCR

Our oligonucleotide primers target DNA sequences of a range of sizes in the human *KRAS* gene around exon 2/Intron 3. The sizes of the amplicons were 67 bp, 145 bp and 320 bp. The reverse primer used was the same for all sizes. Our Q-PCR experiments followed the MIQE guideline (3). Q-PCR amplifications were performed, at least in duplicate, in a 25 µl reaction volume on a CFX96 instrument using the CFX manager software 3.0 (Bio-Rad). Each PCR reaction mixture was composed of 12.5 µl PCR mix (Bio-Rad Super mix SYBR Green), 2.5 µl of each amplification primer (0.3 pmol/µl, final concentration), 2.5 µl PCR-analyzed water, and 5 µl DNA extract. Thermal cycling consisted of a 3 minute Hot-start Polymerase activation denaturation step performed at 95°C, followed by 40 repeated cycles at 95°C for 10 s, then at 60°C for 30 s. Melting curves were obtained by increasing the temperature from 55°C to 90°C, with a plate reading every 0.2°C. Serial dilutions of genomic DNA from the DIFI cell line were used to calibrate the quantifications. Sample concentrations were extrapolated from this standard curve. Negative controls were used in duplicate for each experiment. The efficiency of these primers was assessed using a genomic DIFI cell line as reference. The DNA concentration corresponds to the number of amplicons obtained for each targeted size. Size range fractions were calculated by subtracting the quantity obtained with the larger amplicon from that obtained with the shorter amplicon and dividing by the quantity of the largest amplicon. Because Q-PCR provides a size profile based on the detection of ssDNA fragments, the length is consequently the number of nucleotides. The fraction of cfDNA fragments above 320 nt corresponds to the amount obtained when targeting a sequence of 320 nt. The size distribution was also represented in three fractions: HF, highly fragmented ssDNA (<145 nt); MF, mono-nucleosomal ssDNA fragments (145-320 nt); and WF, weakly fragmented ssDNA (>320 nt). The Q-PCR analyses were carried out blinded with respect to the NGS sequencing data. We estimated the overall fragmentation level by determining a DII (4), which in this study is based on the Q-PCR-based determination of the ratio fragments over 320 bp to those over 67 bp, in the DNA region used for the examination of the fractional distribution.

The experimental validation of the reproducibility, accuracy and sensitivity of the cfDNA quantification has been reported previously (5). They reveal (i), a coefficient of variation of 21.3% of the DNA concentration; and (ii), irrespective of the targeted sequence, no significant difference in DNA concentration when it is calibrated using a standard curve. Nevertheless, to ensure quality control, the quantification of a targeted wild type (WT) *BRAF* sequence was routinely carried out for each sample. Both WT *KRAS* and *BRAF* primer sets were strongly correlated, as indicated by the Spearman analysis

(r ¼ 0.966, $p < 0.001$). The fractional distribution of the plasma cfDNA does not vary much when using nested primers sets targeting four different DNA region (5–7).

Because of the chromatin nature of the cfDNA/nucleosome structure circulating in blood, cfDNA quantification by targeting sequences bigger than 145 bp would be expected to show high variability, as nucleosome positioning along DNA varies greatly. Consequently, determining the cfDNA concentration of amplicons of ~300 bp length, as we did here, would show significant variation in the targeted sequences. One possible solution would be to target a few dozen DNA sequences over 300 bp, and to determine their mean value. This would be misleading, however, because of the consequences of epigenetic events occurring naturally in normal cellular functions: methylation of DNA and histones, for instance, causes nucleosomes condensation; likewise, histone acetylation results in loose packing of nucleosomes, thereby activating gene transcription. Any alteration of this interplay between DNA methylation, gene transcription, and chromatin structure results in nucleosome repositioning and carcinogenesis. Our previous study of the variability of the DII when comparing five different DNA regions or genes showed (i), that the use of nesting the ~60 bp targeted sequence within the ~300 bp targeted sequence used here appears to diminish variability (5, 7); (ii), that a linear repartition of the DII data of *KRAS* and *BRAF* systems is observed; and (iii), that DII values vary up to 4-fold, and cfDNA quantification by targeting sequences over ~300 bp up to 3-fold. Values of the *KRAS* intron region DII as used here correspond to the average among DII values obtained when targeting these 5 DNA regions. Consequently, the proportion of the cfDNA fragments over 320 bp reported here may have a variation coefficient of ~100%. It thus appears to be indicative, not an absolute measurement.

The cfDNA amount over 67 bp does not exactly correspond to the term “total cfDNA concentration”. It is nonetheless very close to being accurate, since the mean % of fragments below 67bp as determined by sequencing was 0.05% and 1.9% for the healthy individuals and ranged from 0.62 to 2.52% and from 1.29% to 4.76% in cancer patients as determined by DSP-S and SSP-S, respectively. Given that these values (1), are determined from sequencing, (2) consequently correspond only to cfDNA size <~1000 bp, and (3) not taking into account the cfDNA over that size (see Results section), the amount of cfDNA over 67bp is >98.1% and >95.2% of the total cfDNA, in healthy and cancer subjects, respectively.

Supplemental Methods Appendix 3

Determination of the cfDNA mutant allele frequency

Mutant allele frequency (MAF) corresponds to the proportion of cfDNA fragments within a plasma extract which bears a targeted mutation. The MAF was determined using the IntPlex assay, which is clinically validated (2). Using 3 mL of plasma, the analysis was carried out by testing 28 different mutations on *KRAS, BRAF* and *NRAS* genes actionable in mCRC management care. The IntPlex allele-specific blocker quantitative PCR (ASB Q-PCR) method was used, as previously described. Intplex design is based on initial studies concerning cfDNA fragmentation and, consequently, targets only short sequences (60-90 bp), with WT targeted sequences located at no more than 300 bp from the mutation. Note, the mutations of the panel are present in ~76% of the mCRC plasma DNA (8). The MAF depends on the number of malignant cells vs the number of non-malignant cells of the tumor microenvironment (lymphocytic, endothelial, stromal cells) and of germinal origin. Targeted sequences and amplicon size are described in Supplemental Figure 10.

References

1. Cook L et al. Does Size Matter? Comparison of Extraction Yields for Different-Sized DNA Fragments by Seven Different Routine and Four New Circulating Cell-Free Extraction Methods [Internet]. *Journal of Clinical Microbiology* 2018;56(12). doi:10.1128/JCM.01061-18

2. Thierry AR et al. Clinical validation of the detection of KRAS and BRAF mutations from circulating tumor DNA. *Nat Med* 2014;20(4):430–435.

3. Bustin SA et al. The MIQE Guidelines: Minimum Information for Publication of Quantitative Real-Time PCR Experiments. *Clinical Chemistry* 2009;55(4):611–622.

4. Mouliere F et al. High Fragmentation Characterizes Tumour-Derived Circulating DNA. *PLOS ONE* 2011;6(9):e23418.

5. Mouliere F, El Messaoudi S, Pang D, Dritschilo A, Thierry AR. Multi-marker analysis of circulating cell-free DNA toward personalized medicine for colorectal cancer. *Mol Oncol* 2014;8(5):927–941.

6. Serpas L et al. Dnase1l3 deletion causes aberrations in length and end-motif frequencies in plasma DNA. *PNAS* 2019;116(2):641–649.

7. Mouliere F et al. Circulating Cell-Free DNA from Colorectal Cancer Patients May Reveal High KRAS or BRAF Mutation Load. *Translational Oncology* 2013;6(3):319.

8. Thierry AR et al. Clinical utility of circulating DNA analysis for rapid detection of actionable mutations to select metastatic colorectal patients for anti-EGFR treatment. *Ann Oncol* 2017;28:2149–2159.
